# Dynamic Responses of Barley Root Succinyl-Proteome to Short-Term Phosphate Starvation and Recovery

**DOI:** 10.3389/fpls.2021.649147

**Published:** 2021-03-31

**Authors:** Juncheng Wang, Zengke Ma, Chengdao Li, Panrong Ren, Lirong Yao, Baochun Li, Yaxiong Meng, Xiaole Ma, Erjing Si, Ke Yang, Xunwu Shang, Huajun Wang

**Affiliations:** ^1^Gansu Provincial Key Lab of Aridland Crop Science/Gansu Key Lab of Crop Improvement and Germplasm Enhancement, Lanzhou, China; ^2^Department of Crop Genetics and Breeding, College of Agronomy, Gansu Agricultural University, Lanzhou, China; ^3^Western Barley Genetics Alliance, College of Science, Health, Engineering and Education, Murdoch University, Murdoch, WA, Australia; ^4^Department of Botany, College of Life Sciences and Technology, Gansu Agricultural University, Lanzhou, China

**Keywords:** Pi stress, *Hordeum vulgare* L., germplasm, metabolism, succinylated protein

## Abstract

Barley (*Hordeum vulgare* L.)—a major cereal crop—has low Pi demand, which is a distinct advantage for studying the tolerance mechanisms of phosphorus deficiency. We surveyed dynamic protein succinylation events in barley roots in response to and recovery from Pi starvation by firstly evaluating the impact of Pi starvation in a Pi-tolerant (GN121) and Pi-sensitive (GN42) barley genotype exposed to long-term low Pi (40 d) followed by a high-Pi recovery for 10 d. An integrated proteomics approach involving label-free, immune-affinity enrichment, and high-resolution LC-MS/MS spectrometric analysis was then used to quantify succinylome and proteome in GN121 roots under short-term Pi starvation (6, 48 h) and Pi recovery (6, 48 h). We identified 2,840 succinylation sites (Ksuc) across 884 proteins; of which, 11 representative Ksuc motifs had the preferred amino acid residue (lysine). Furthermore, there were 81 differentially abundant succinylated proteins (DFASPs) from 119 succinylated sites, 83 DFASPs from 110 succinylated sites, 93 DFASPs from 139 succinylated sites, and 91 DFASPs from 123 succinylated sites during Pi starvation for 6 and 48 h and during Pi recovery for 6 and 48 h, respectively. Pi starvation enriched ribosome pathways, glycolysis, and RNA degradation. Pi recovery enriched the TCA cycle, glycolysis, and oxidative phosphorylation. Importantly, many of the DFASPs identified during Pi starvation were significantly overexpressed during Pi recovery. These results suggest that barley roots can regulate specific Ksuc site changes in response to Pi stress as well as specific metabolic processes. Resolving the metabolic pathways of succinylated protein regulation characteristics will improve phosphate acquisition and utilization efficiency in crops.

## Introduction

Phosphorus (P)-absorbed in the inorganic form of phosphate (Pi) by plants-is a limiting factor for plant growth and crop production worldwide (Mora-Macías et al., 2017; Pan et al., 2019). Pi is an essential constituent of fundamental molecules, including nucleic acids, ATP, and membrane phospholipids, and its low availability in soil often results in Pi deficiency in plants. In general, crops assimilate about 20–30% of the Pi from applied P fertilizer (Lopez-Arredondo et al., 2014). The application of large quantities of P fertilizers in soil is not only unsustainable due to the gradual depletion of phosphate rock but also causes serious water and soil pollution due to the unused P (Cordell et al., 2009; Kochian, 2012; Pan et al., 2019). Understanding the molecular mechanism of the phosphate starvation response (PSR) in plants and improving Pi acquisition and utilization efficiency are critical for developing Pi-efficient crop varieties.

Plants have evolved a set of adaptive responses to improve Pi uptake by roots and recirculate Pi from storage compartments and senescent tissues under Pi starvation (Lopez-Arredondo et al., 2014; Péret et al., 2014; Lambers et al., 2015; Pan et al., 2019; Oldroyd and Leyser, 2020). These measures include modifying root system architecture (RSA), secreting organic acids and Pi-releasing enzymes, regulating the expression and activity of Pi transporters, and reprogramming associated metabolism pathways (Lan et al., 2018).

The Pi-starvation response in *Arabidopsis* is divided into locally and systemically regulated groups according to external and internal Pi status, respectively. The local regulation response mainly involves root developmental adaptations, whereas internal Pi homeostasis is regulated at the systemic level (Rouached et al., 2010; Thibaud et al., 2010). Modifications to RSA, including primary root growth inhibition, lateral root formation and elongation, and root hair proliferation, improve the root surface area for exploration in shallow soil and promote topsoil foraging upon Pi depletion (Mora-Macías et al., 2017). In many species, these alterations to RSA are associated with alternative strategies to cope with Pi limitations.

Over the past decade, Pi sensing and signal transduction and the key roles of several Pi-starvation response (PSR) genes in regulating RSA upon Pi starvation have been defined and reviewed (Svistoonoff et al., 2007; Péret et al., 2014; Puga et al., 2017; Wang et al., 2018). Post-translational modifications (PTMs) are dynamic and reversible protein processes that modulate the activity of target proteins by regulating their stability, activity, localization, and signaling pathway (Mann and Jensen, 2003; Rao et al., 2014), including characterized phosphorylation, ubiquitination, methylation glycosylation, and carbonylation, and newly defined succinylation, nitrosylation and crotonylation (Xu W. et al., 2017). Extensive evidence has revealed that PTMs play an essential role in regulating RSA under Pi stress. Plant protein PTMs via ubiquitination is well-characterized and mainly involves the auxin signaling pathway [e.g., SIZ1 (Miura et al., 2005), TIR1 (Pérez-Torres et al., 2008), MAX2 (Mayzlish-Gati et al., 2012), BES1 (Singh et al., 2014), PIN2 (Leitner et al., 2012)] and autophagy activation [e.g., NLA (Kant et al., 2011), LPR2 (Svistoonoff et al., 2007), UBP14 (Li et al., 2010), OTU5 (Suen et al., 2018)] to regulate RAS remodeling copy with Pi starvation. Only a few genes encoding proteins involved in RSA are represented in the other PTM types. Pi deficiency enhanced phosphoenolpyruvate carboxylase (PEPC) activity in *Lupinus albus* roots (Johnson et al., 1996). Michael et al. revealed that *in vivo* PEPC activation via phosphorylation contributes to organic acid synthesis and exudation that dominates carbon metabolism in proteoid roots in Pi-deficient harsh hakea (*Hakea prostrata*) (Shane et al., 2013). Indirect evidence indicated that phosphorylation of transcription factors by a novel P-starvation tolerance 1 (PSTOL1) gene that encodes PSI protein kinase regulated gene expression to enhance early root growth in rice under P-deficient soils (Gamuyao et al., 2012). Histone deacetylase HDA19 controls the epidermal cell length of roots, and regulates genes encoding SPX domain-containing proteins and those involved in membrane lipid remodeling during acclimation to Pi deficiency (Chen et al., 2015). The functional significance of PTMs in the RSA response to Pi starvation and the intricate crosstalk between PTM types needs to be investigated.

Barley (*Hordeum vulgare* L.) is a major cereal crop grown worldwide that has distinct advantages as a model species for studying the mechanisms of tolerance to P deficiency due to low Pi demand. Our previous study revealed that barley genotype GN121 has high phosphorus utilization efficiency (PUE); low P conditions significantly enriched differentially expressed genes involved in P metabolism in GN121 (Ren et al., 2018), but the regulatory role of PTMs in RSA of barley in response to Pi limitations is unknown. Antibody-based affinity enrichment and highly sensitive mass spectrometry can be used to identify most expressed proteins and for the global analysis of protein succinylation with good accuracy and reproducibility (Aebersold and Mann, 2016; Zhou et al., 2018).

In the present study, we present an integrated whole-genome quantitative succinylation (Ksuc) proteomic approach to compare the response to Pi starvation in high PUE barley roots during the short-term and during a recovery course. Our results reveal a very distinct global “omic” quantitative profile and succinylated proteins in response to Pi stress. Furthermore, our dynamic proteomic profile identified the regulatory Pi starvation and recovery response pathways, as well as distinct features of succinylation in response to Pi stress.

## Materials and Methods

### Materials and Pi Starvation Treatment Conditions

Two spring barley genotypes—GN121 (low-Pi-tolerant) and GN42 (low-Pi-sensitive)—were used (Ren et al., 2016). Seeds of GN121 and GN42 were obtained from Gansu Agricultural University (Lanzhou, China). These seedlings and plant growth in hydroponic culture conditions are described elsewhere under long-day (16 h light/8 h dark cycle) conditions at a temperature 20 ± 5°C with 50–70% relative humidity and irradiation intensity of approximately 300 μmol m^−2^ s^−1^ (Ren et al., 2018). Briefly, seeds were surface sterilized and pre-germinated; on day 10, after removing the endosperms, the seedlings were transplanted into a modified Hoagland hydroponic solution containing 0.39 mM KH_2_PO_4_ (high Pi, +Pi) or 0.039 mM KH_2_PO_4_ (low Pi, –Pi).

For long-term Pi starvation, GN121 and GN42 were grown under low Pi for 40 d and then resupplied with high Pi for 10 d. Control plants were grown for 50 d with high Pi. The nutrient solutions were renewed weekly to avoid nutrient depletion below 70% of the initial concentration. Plants were sampled every 10 days to monitor root development. Five biological replicates were collected for each time point. The fresh roots from 10 individual plants of each biological replicate were scanned using an EPSON1680 scanner (Epson, Long Beach, CA, USA) at 300 dpi. The scans were analyzed using WinRHIZO software (Regent Instruments Inc., Quebec, ON, Canada) to quantify total root length, surface area, and volume, and were then used to determine fresh weight of the roots.

For short-term Pi starvation, low-Pi-tolerant GN121 was grown under low Pi for 48 h and then resupplied with high Pi for 48 h. Roots were harvested on four occasions in three biological replicates for protein extraction: (1) Pi-starvation phase, 6 h and 48 h after exposure to low Pi; (2) Pi-recovery phase, 6 and 48 h after being resupplied with high Pi.

### Protein Extraction

Proteins were extracted as described elsewhere (Zhou et al., 2018). Briefly, the root samples were ground in liquid nitrogen and then homogenized in lysis buffer containing 8 M urea, 1% Triton-100, 10 mM dithiothreitol, and 1% protease inhibitor cocktail. The mixtures were sonicated three times on ice before removing the remaining debris by centrifugation at 20,000 *g* for 10 min at 4°C. Soluble proteins were precipitated with cold 20% trifluoroacetic acid (TCA) for 2 h at −20°C. After centrifugation at 12,000 *g* for 10 min at 4°C, the supernatant was discarded. The protein precipitates were washed three times with ice-cold acetone. The proteins were dissolved in 8 M urea, and the concentrations were determined using a BCA (Pierce, Bonn, Germany) kit according to the manufacturer's instructions.

### Immunoblot

Proteins were isolated from the roots of GN121 in the Pi-starvation phase (0, 3, 6, 12, 24, 48, 72 h, 5 d, and 7 d) and Pi-recovery phase (3, 6, 12, 24, 48 h and 72 h). For immunoblotting, 20 μg protein from each sample was separated using 12% SDS-PAGE and electroblotted onto polyvinylidene fluoride (PVDF) membrane. The blot was detected by the pan anti-succinyllysine antibody (1:1000 dilution; PTM Biolabs, Hangzhou, China), washed extensively with PBS buffer plus 1% Tween 20, and then probed with alkaline phosphatase conjugated goat secondary anti-mouse IgG peroxidase antibody (1:5000 dilution; Thermo Fisher Scientific, Pierce, USA).

### Trypsin Digestion

The protein solution was reduced by adding 10 mM DTT and incubating at 37°C for 1 h. Proteins were then alkylated with 20 mM iodoacetamide at room temperature for 45 min in the dark. For trypsin digestion, the protein was diluted by adding 100 mM TEAB to urea (<2 M) before adding trypsin at 1:50 trypsin:protein mass ratio for the first digestion overnight and 1:100 trypsin:protein mass ratio for the second 4 h digestion.

### Affinity Enrichment

For succinylation, the tryptic peptides were dissolved in IP buffer (100 mM NaCl, 1 mM EDTA, 50 mM Tris-HCl, 0.5% NP-40, pH 8.0). The supernatant was transferred to pre-washed antibody beads (PTM Biolabs, Hangzhou, China) to bind Ksuc peptides. The mixtures were gently shaken at 4°C overnight. The beads were then washed four times with IP buffer and twice with ddH_2_O. Finally, 0.1% trifluoroacetic acid was added three times to elute the bound peptides, which were vacuum-dried. The resulting peptides were desalted with C18 ZipTips (Millipore), according to the manufacturer's instructions for LC-MS/MS analysis.

### LC-MS/MS Spectrometric Analysis

The tryptic peptides were dissolved in solvent A (0.1% formic acid, 2% acetonitrile/ in water), directly loaded onto a PTM Biolabs-made reversed-phase analytical column (ReproSil-Pur C18-AQ, 1.9 μm; Dr Maisch; 25 cm length, 75 μm i.d.). Peptides were separated with a gradient from 6 to 22% solvent B (0.1% formic acid in acetonitrile) over 70 min, 22 to 32% in 14 min and climbing to 80% in 3 min then holding at 80% for the last 3 min, all at a constant flow rate of 300 nL/min on a nanoElute UHPLC system (Bruker Daltonics).

The succinylation peptides were also dissolved in solvent A (0.1% formic acid, 2% acetonitrile/ in water), directly loaded onto a PTM Biolabs-made reversed-phase analytical column (ReproSil-Pur C18-AQ, 1.9 μm; Dr Maisch; 25 cm length, 75 μm i.d.). Peptides were separated with a gradient from 6 to 22% solvent B (0.1% formic acid in acetonitrile) over 43 min, 22–30% in 13 min and climbing to 80% in 3 min then holding at 80% for the last 3 min, all at a constant flow rate of 450 nL/min on a nanoElute UHPLC system.

The peptides were subjected to CaptiveSpray source followed by the timsTOF Pro (Bruker Daltonics) mass spectrometry. The electrospray voltage applied was 1.60 kV. Precursors and fragments were analyzed at the TOF detector, with a MS/MS scan range from 100 to 1700 m/z. The timsTOF Pro was operated in parallel accumulation serial fragmentation (PASEF) mode. Precursors with charge states 0–5 were selected for fragmentation, and 10 PASEF-MS/MS scans were acquired per cycle. The dynamic exclusion was set to 30 s.

### Database Search

The resulting MS/MS data were processed using the MaxQuant search engine (v.1.6.6.0; Max Plank Institute of Biochemistry, Germany). Group-specific parameters and fractions were defined for the whole proteome and succinyllysine-enriched samples, respectively. Tandem mass spectra were searched against the *Hordeum vulgare* L. protein database (https://webblast.ipk-gatersleben.de/barley_ibsc/downloads; 39,743 protein entries, downloaded at July 23th, 2019) with the following parameters: (1) Trypsin/P specified as the cleavage enzyme allowing up to two missing cleavages; (2) mass tolerance for precursor ions set as 20 ppm in the first and main search, and 0.02 Da for fragment ions; (3) carbamidomethyl on Cys as a fixed modification, and (4) oxidation on Met and acetylation on the protein N-terminus specified as variable modifications. For succinylation peptides, succinylation on Lys was also specified as variable modifications. False discovery rate thresholds for protein, peptide, and modification sites were adjusted to <1%. The minimum peptide length was set at 7. The site localization probability was set as >0.75. The minimum score for modified peptides was set >40.

Label-free quantification (LFQ) was based on extracted ion currents (XICs) of peptides, and the intensity-based absolute quantification (iBAQ) in MaxQuant was used to quantify succinylated protein abundance (Cox et al., 2014).

### Bioinformatics Annotation Analysis

For protein LFQ, the iBAQ approach was used to calculate protein abundance based on the extracted ion currents (XICs) of peptides (Schwanhäusser et al., 2011). Only the abundance succinylated peptides with consistent fold-changes in at least two of the three (not a NaN) replicates were counted. The significance of the change in abundance among samples was evaluated as differentially expressed by a fold-change in abundance > 1.5 with a *P*-value < 0.05 according to one-way analysis of variance followed by Student's *t*-test.

The Kyoto Encyclopedia of Genes and Genomes (KEGG, http://www.kegg.jp/) database was used to annotate the significantly enriched protein pathways with a corrected *P* < 0.05. For each GO category, a two-tailed Fisher's exact test was used to calculate the enrichment of each identified protein against the GO database (*P* < 0.05). InterProScan (http://www.ebi.ac.uk/interpro/) was used for protein domain annotation. Conserved amino-acid sequence motifs of succyl-21-mers (ten amino acids upstream and downstream of the site) were analyzed using Motif-X (http://meme-suite.org/tools/momo) with *P* < 0.000001. Furthermore, all protein sequences in the barley database were used as the background database parameter. A subcellular localization predication soft WoLF PSORT (http://www.genscript.com/psort/wolf_psort.html) was used to predict subcellular localization of the protein. The R package Mfuzz was used for hierarchical clustering analyses (HCL) of the differentially succinylated sites based on the relative succinylation intensity. Protein–protein interaction (PPI) network analysis was obtained using STRING software (v.10.5) according to a confidence score >0.7 and visualized by Cytoscape software (version 3.6.1). A graph-theoretical clustering algorithm, molecular complex detection (MCODE), was used to analyze densely connected regions.

## Results

### Plant Growth Response to Changes in Plant Pi Status

**Figure 1:**
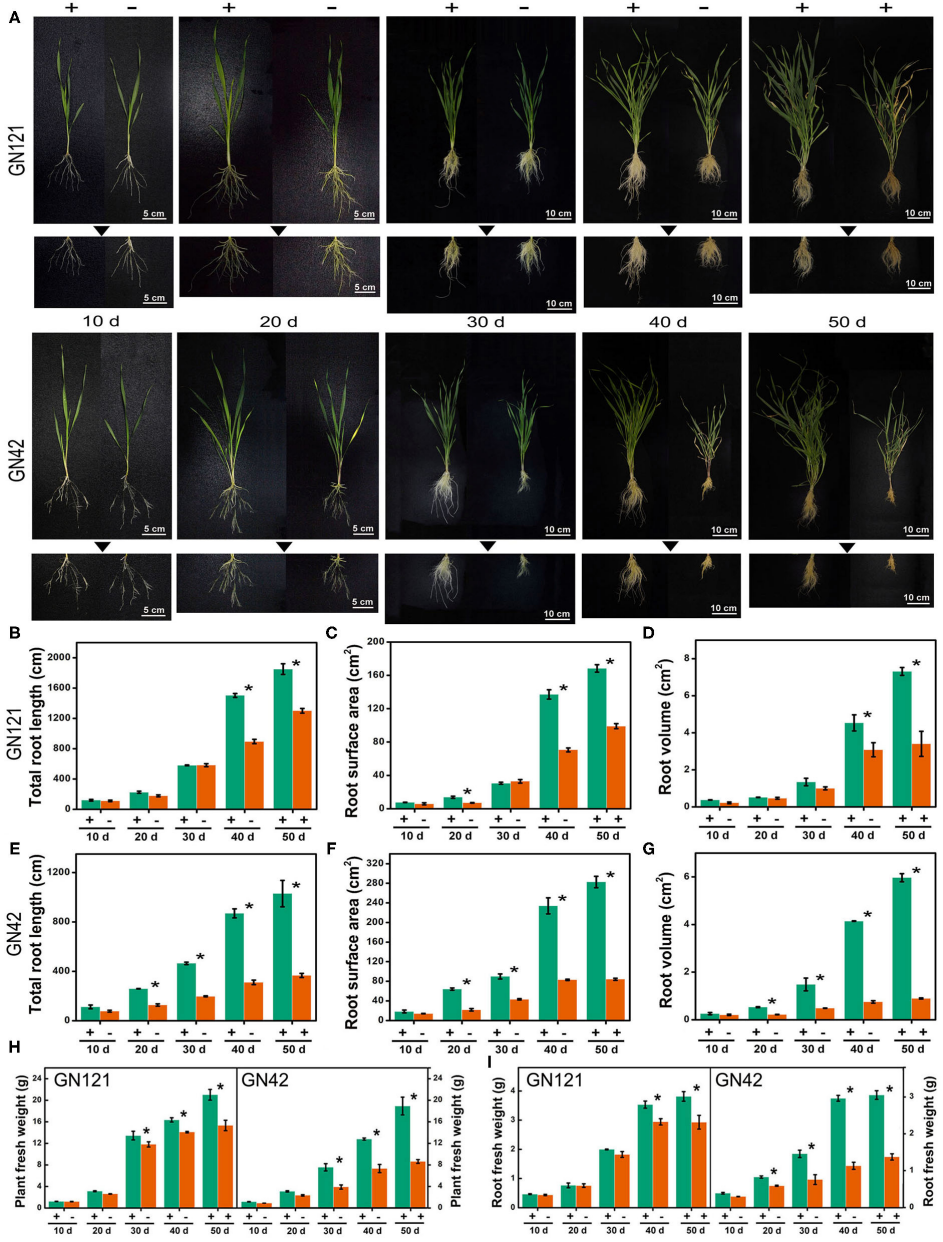
Root characteristics of two barley lines with different low-Pi tolerance in response to Pi starvation and recovery. **(A)** GN121 (Pi-tolerant) and GN42 (Pi-sensitive) seedlings grown in normal P (+, 0.39 mM Pi) and low P (–, 0.039 mM Pi) for 10, 20, 30, or 40 d, followed by recovery P (+, 0.39 mM P) for 10 d; **(B)** total root length, **(C)** root surface area, and **(D)** root volume of GN121 seedlings; **(E)** total root length, **(F)** root surface area, and **(G)** root volume of GN42 seedlings; **(H,I)** are plant and root fresh weights of GN121 and GN42 seedlings, respectively. Data are means ± SD (*n* = 5); * Indicates significant differences (one-way ANOVA, Duncan, *P* ≤ 0.05) between normal and Pi treatments.

Plant root architecture adjustment is a crucial adaptive response during Pi deficit. For long-term Pi starvation, barley seedlings of GN121 (Pi-tolerant) and GN42 (Pi-sensitive) were submitted to a range of Pi regimes, including grown under low Pi for 40 d and followed by a recovery with high Pi for 10 d. We compared the root architecture, including root length, surface area, volume, and biomass, of two low-Pi tolerance barley genotypes under low P stress to identify changes triggered by Pi starvation (Figure 1). Phosphate depletion changed the RSA of the two barley genotypes, which was more evident with the continuous low-Pi stress. Low-Pi stress significantly decreased seedling root length, surface area, volume, and biomass, relative to the high-Pi control, more so in the low-Pi-sensitive GN42 than the low-Pi-tolerant GN121 (Figure 1). The Pi-recovery phase induced a partial recovery in root development, especially in GN121, indicating that it is an ideal genotype for studying root developmental adaptations and identifying important regulators under low Pi.

### Protein Lysine Succinylation in GN121 Roots Under Different Pi Conditions

**Figure 2:**
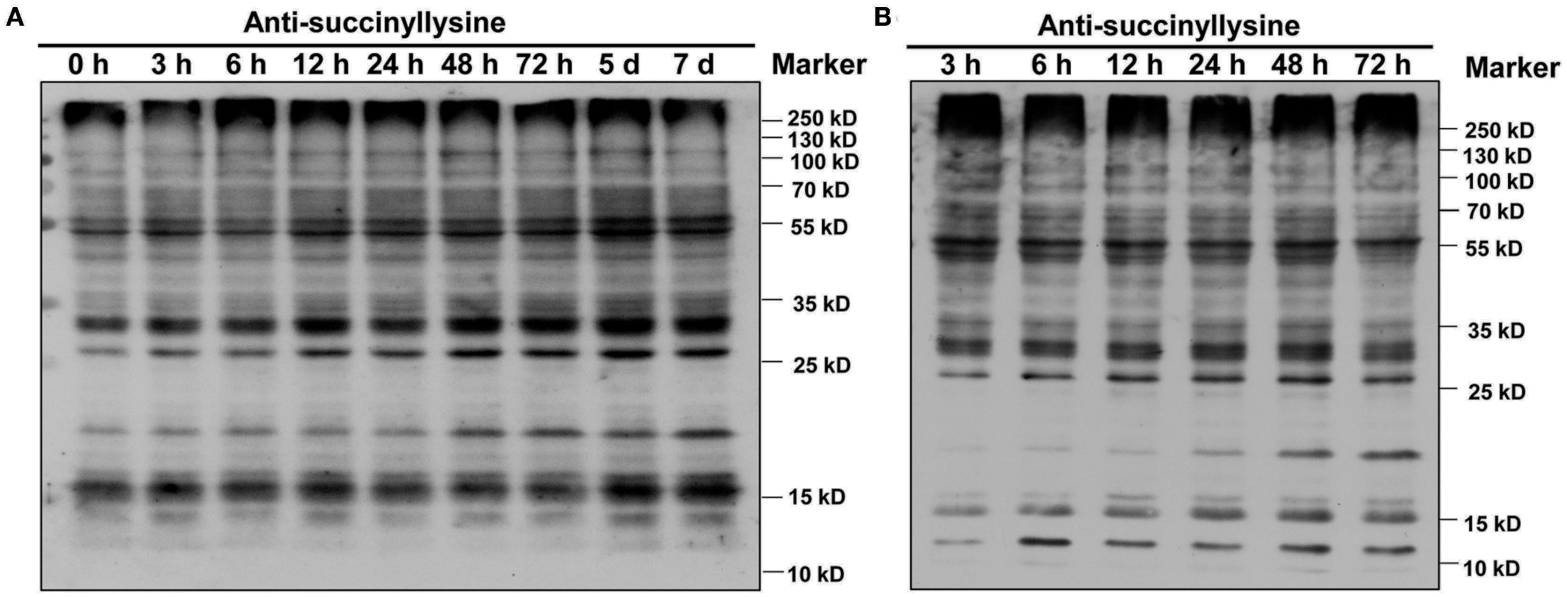
Dynamics of protein succinylation in roots of GN121 seedlings under Pi starvation and recovery using the anti-suc-lysine antibody. For immunoblot results, proteins were collected from **(A)** low-Pi (0.039 mM Pi) at 0, 3, 6, 12, 24, 48, 72 h, 5 d, and 7 d, and **(B)** Pi recovery (0.39 mM Pi) at 3, 6, 12, 24, 48, and 72 h. The same amount of protein (25 μg per lane) was loaded in each panel.

We investigated global succinylated protein-level changes under different Pi conditions. Proteins were isolated from the roots of GN121 under Pi starvation (0, 3, 6, 12, 24, 48, 72 h, 5 d, and 7d) and Pi recovery (3, 6, 12, 24, 48, and 72 h). The protein samples were analyzed using highly sensitive and lysine succinylation-specific pan-antibodies. The immunoblot results revealed succinyllysine proteins with a wide range of molecules that differed between the Pi-starvation and Pi-recovery phases (Figure 2). Importantly, roots during Pi starvation displayed more signals 10–25 kD and less specific signals among 55–100 kD compared with Pi recovery (Figure 2). The overall succinylated protein levels increased with the duration of Pi starvation/recovery, relative to the 0 h control, suggesting that lysine succinylation regulates different protein functions and succinylation levels at different times during Pi starvation/recovery. The most significant differences in succinyllysine levels occurred within 48 h of Pi starvation and Pi recovery, relative to the 0 h control (Figure 2).

**Figure 3:**
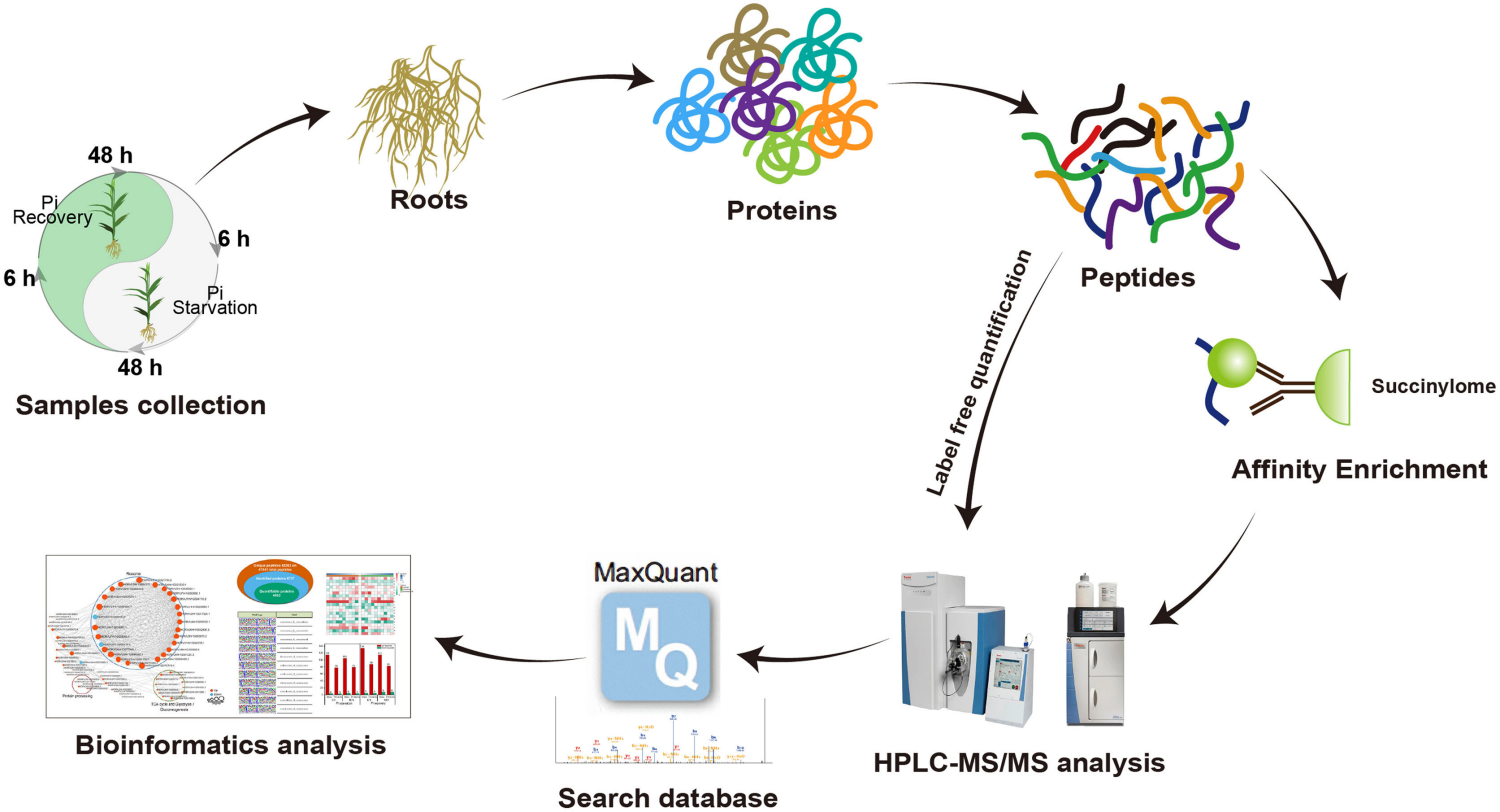
Workflow used to analyze lysine succinylation in seedling roots in response to Pi starvation and recovery.

To comprehensively assess lysine succinylome dynamics in roots of Pi-tolerant barley variety GN121 under different Pi conditions, we designed a short-term Pi starvation approach with a Pi-starvation phase (6, 48 h) and Pi-recovery phase (6, 48 h). An integrated proteomics approach involving label-free, immune-affinity enrichment, and high-resolution LC-MS/MS spectrometric analysis was used to quantify the succinylome and proteome of GN121 roots under different Pi levels to investigate the changes in specific increased or decreased and succinylated proteins. The general experimental workflow is shown in Figure 3. The mass spectrometry data of the succinylome and proteome have been deposited at the ProteomeXchange with dataset identifier PXD022052 and PXD022053, respectively.

### Identification and Characterization of Succinylome in Roots During Pi Starvation and Recovery Phases

**Figure 4:**
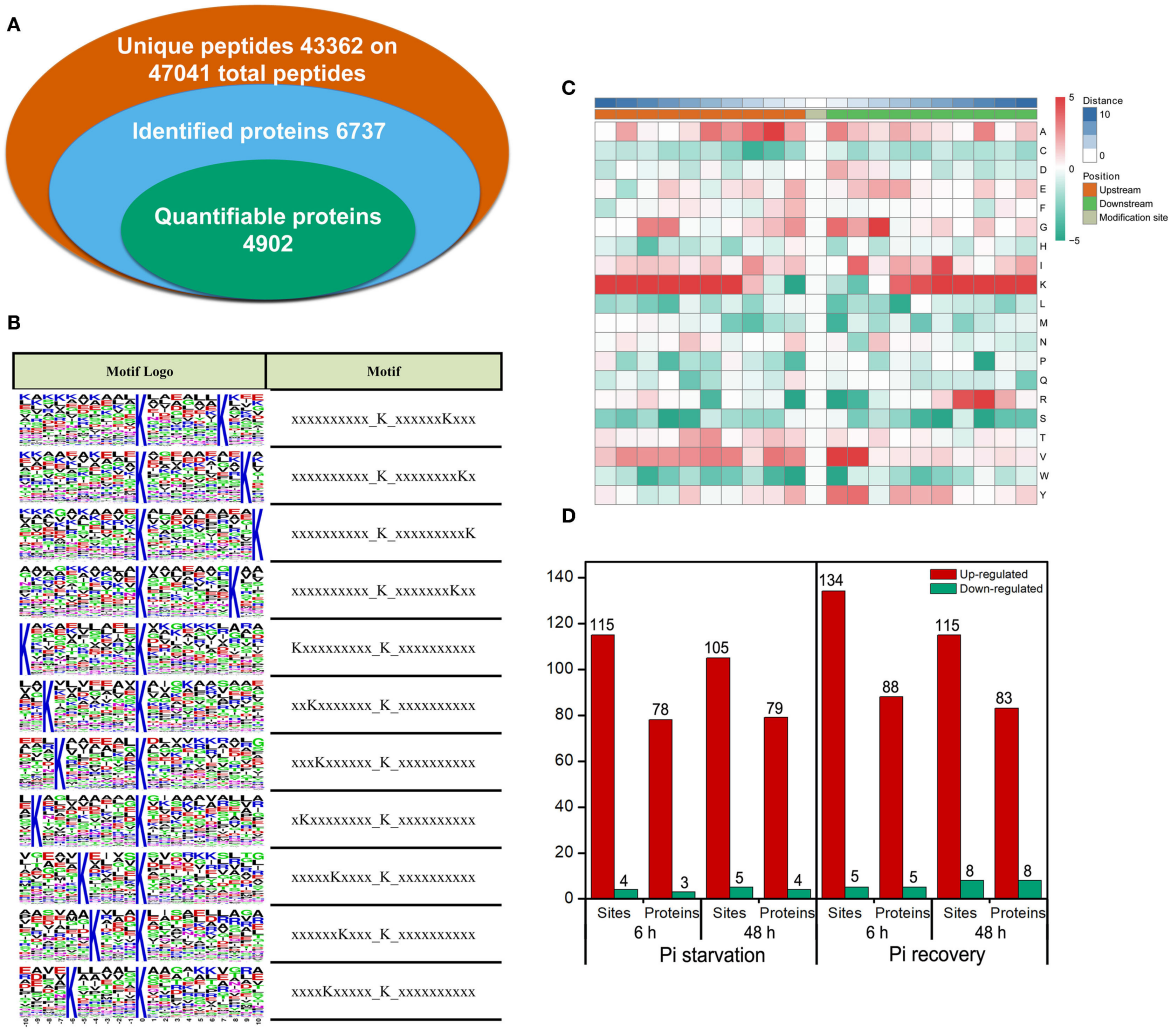
Properties of the succinylated peptides in barley roots. **(A)** Summary of the succinylome identified and quantified; **(B)** motif analysis of identified succinylated sequence by Motif-X software. The motifs with high significance (*P* < 0.000001) are shown; **(C)** position-specific amino acid composition around the succinylation sites. The –log10 (Fisher's exact test *P*-value) for every amino acid in each position (from −10 to +10) is shown; **(D)** overview of the differentially succinylated sites and proteins (fold change > 1.5, *P* < 0.05).

We identified 2,840 Ksuc sites across 884 proteins with a high score (>60) and a high confidence localization score (>0.75), of which 2,137 succinylated-lysine sites from 697 proteins were quantified (Figure 4A). To understand the regulation and amino acid residue preference at the sites surrounding the succinylated lysine, we carried out succinylation site motif analysis by examining the sequences from −10 to +10 of the 2,840 succinylation sites using the Motif-X program. There were 11 distinguished motifs identified—all with the preferred amino acid residue of lysine—including K_−10/−9/−8/−7/−6/−5/−4_Ksuc and K_+10/+9/+8/+7_Ksuc (Figure 4B). The frequency of amino acid residues flanking succinylated lysine was analyzed to investigate the enrichment or depletion of various amino acids (Figure 4C). Lysine (K) from −4 to −10 and +4 to +10 positions, valine (V) from +1 to +2 positions, arginine (R) from −7 to −8 positions, and alanine (A) from +2 to +3 positions were preferred, and these patterns agreed with the identified conserved motifs reported in this study. After setting a quantification ratio of >1.5 and *P* < 0.05 as cutoff, we identified difffrentially abundant succinylated proteins (DFASPs) and sites within each group. Compared with the control, there were 81 DFASPs from 119 succinylated sites during Pi starvation (6 h), 83 DFASPs from 110 succinylated sites during Pi starvation (48 h), 93 DFASPs from 139 succinylated sites during Pi recovery (6 h), 91 DFASPs from 123 succinylated sites during Pi recovery (48 h) (Figure 4D). The average degree of succinylation in these DFASPs ranged from 1.33 to 1.49, indicating that most of the succinylated proteins contained only one Ksuc site and that one Ksuc site can affect protein function (Supplementary Table 1). Moreover, at least five heavily succinylated proteins contained ≥4 Ksuc sites, including adenosylhomocysteinase (HORVU2Hr1G110120.3, SAHase, five sites), glyceraldehyde-3-phosphate dehydrogenase C2 (HORVU6Hr1G054520.3, GAPDH C2, four sites), and mitochondrial ADP/ATP carrier protein (HORVU6Hr1G070780.1) during Pi starvation, and ATP synthase subunit beta (HORVU1Hr1G083840.2, four sites), fructose-bisphosphate aldolase 2 (HORVU3Hr1G088540.1, four sites), and the mitochondrial ADP/ATP carrier protein (HORVU6Hr1G070780.1) during Pi recovery (Supplementary Table 1). The mitochondrial ADP/ATP carrier protein catalyzes the ADP import from the cytosol and ATP export from the mitochondrial matrix (Dahout-Gonzalez et al., 2006), which had the most succinylated-lysine sites, being five, six, six, and seven in a single protein during Pi starvation (6 h), Pi starvation (48 h), Pi recovery (6 h), and Pi recovery (48 h), respectively (Supplementary Table 1).

SAHase serves as a major regulator of SAM (S-adenosylmethionine)-dependent biological DNA methylation reactions by removing the SAH (S-adenosylhomocysteine) product involved in the ethylene biosynthetic pathway, which regulates many aspects of growth and development (Ravanel et al., 1998). Glyceraldehyde 3-phosphate dehydrogenase (GAPDH) plays a key regulatory function in the glycolysis pathway but may be a multifunctional protein involved in various cellular processes, such as DNA repair and regulation of redox homeostasis (Yuan et al., 2019a). The accumulation of ATP synthase subunit beta (Atp2), as a negative plant cell death regulator, enabled roots to grow rapidly during Pi recovery (Chivasa et al., 2011). Fructose-bisphosphate aldolase is another glycolytic enzyme, which catalyzes an aldol cleavage of fructose-1, 6-bisphosphate to dihydroxyacetone-phosphate and glyceraldehyde 3-phosphate and a reversible aldol condensation; its activity increases in rice roots treated with gibberellin (GA) (Konishi et al., 2004) and under salt stress (Long et al., 2016). However, the role of succinylation modification on these proteins in the root response to Pi nutrition is unknown.

**Figure 5:**
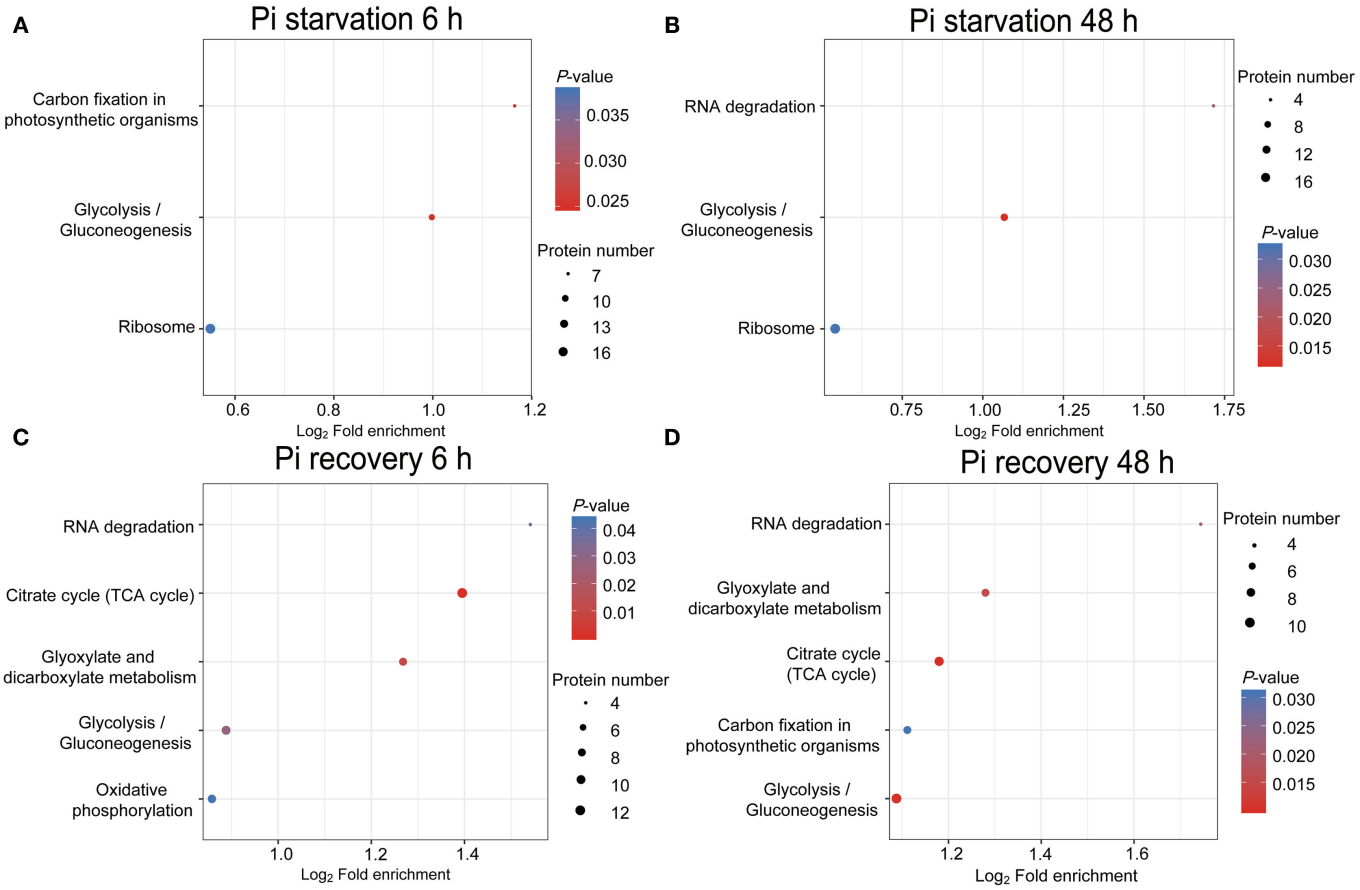
KEGG pathway enrichment analysis of DFASPs in roots under Pi starvation at 6 h **(A)** and 48 h **(B)** and Pi recovery at 6 h **(C)** and 48 h **(D)**.

Enrichment analysis (*P* < 0.05) using the KEGG, GO, and InterPro domain was undertaken to investigate the possible roles of these DFASPs. The KEGG pathway enrichment analysis showed a broad distribution of these DFASPs at different Pi levels (Supplementary Table 2). The ribosome and glycolysis/gluconeogenesis pathways were the most enriched ones during Pi starvation, and the citrate cycle (TCA cycle), glycolysis/gluconeogenesis, and glyoxylate and dicarboxylate metabolism pathways were the most enriched ones during Pi recovery (Figure 5).

The GO biological process category annotation indicated a wide range of cellular and metabolic processes that were susceptible to regulation by succinylation (Supplementary Figure 1; Supplementary Table 3). The subcellular localization prediction revealed about 90% of the lysine-succinylated proteins were located in chloroplasts, cytoplasm, mitochondria, and the nucleus (Supplementary Figure 2). Interestingly, the enrichment analysis of the InterPro domain showed that lysine-succinylated substrates-ATP synthase alpha/beta family, beta-barrel domain, and ATP synthase alpha/beta family, nucleotide-binding domain—were enriched during Pi starvation and Pi recovery (Supplementary Figure 3; Supplementary Table 4). These results indicate that succinylation differs during Pi starvation and recovery, and the enriched succinylation is vital for the regulation of energy metabolism in the Pi-starvation response.

### Succinylome Dynamics in Roots During Pi Starvation and Recovery Phases

**Figure 6:**
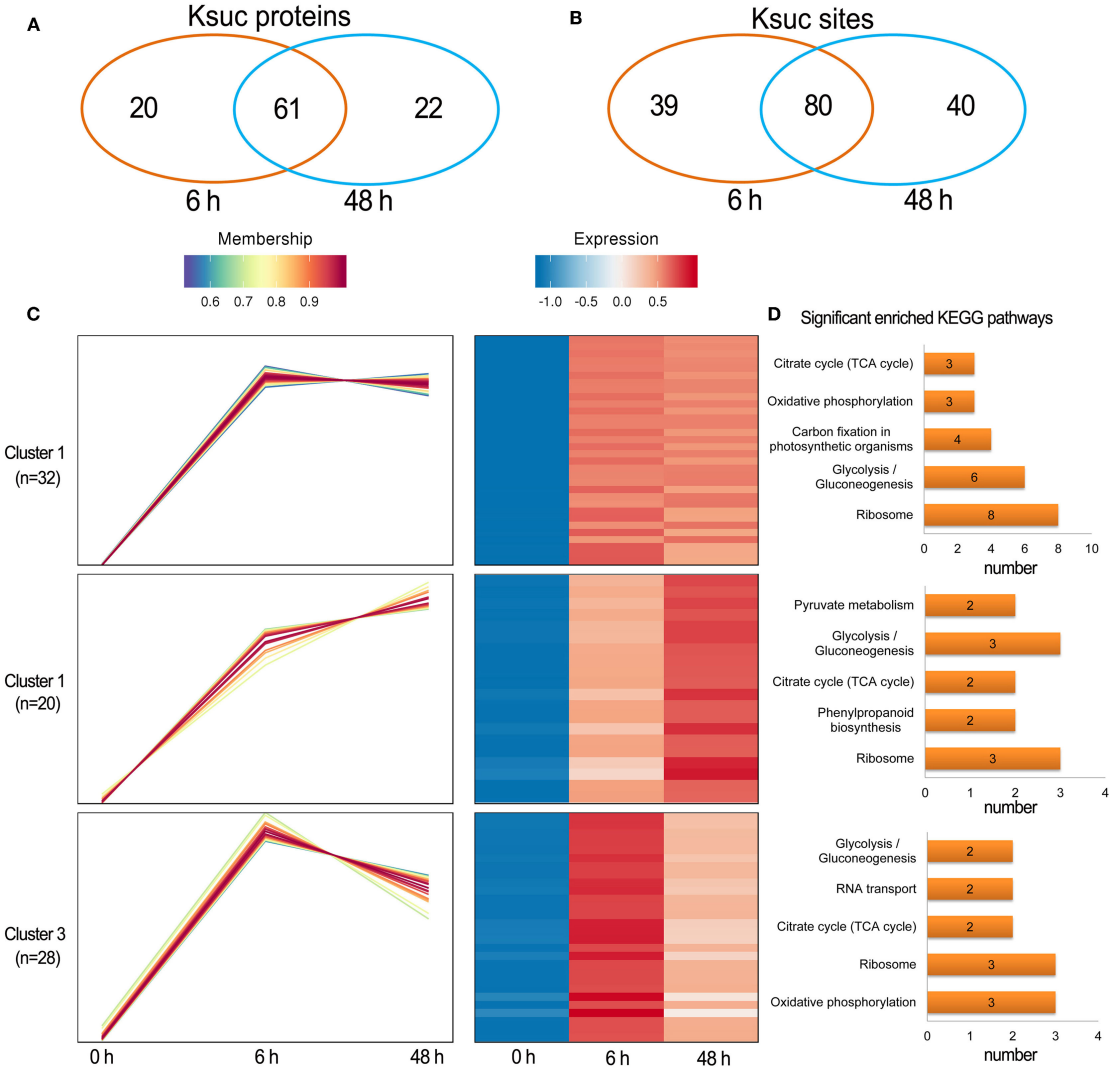
Summary of the differentially succinylated proteins in response to Pi starvation. **(A,B)** Venn diagram of difffrentially abundant succinylated proteins (sites) in response to Pi starvation; **(C)** functional clustering analyses (HCL) of the differentially succinylated sites based on the relative succinylation intensity, relative to the control. Cluster identification and number of profiles included in each cluster are indicated on the left. A detailed view of individual profiles is in Supplementary Figure 6; **(D)** KEGG pathway enrichment analysis of DFASPs in each cluster.

Quantitative succinylome profiling was undertaken to investigate the effects of lysine succinylation on Pi starvation and recovery. The profiling identified 78 increased DFASPs with 115 succinylation sites and three decreased DFASPs with four succinylation sites in the Pi starvation (6 h) treatment, and 79 increased DFASPs with 105 succinylation sites and four decreased DFASPs with five succinylation sites in the Pi starvation (48 h) treatment (Figure 4). The increased DFASPs were mainly involved in the glycolysis/gluconeogenesis, ribosome, and RNA degradation pathways (Supplementary Table 2; Figure 5). A Venn diagram of DFASPs was constructed (Figures 6A,B), which showed 61 increased DFASPs with 80 succinylation sites co-expressed at the stage of Pi deficiency (Pi-responsive DFASPs), which were associated with the ribosome and glycolysis/gluconeogenesis pathways (Supplementary Table 5).

The hierarchical clustering analysis (HCL) divided the Pi-responsive DFASPs into three clusters (Figure 6C). Cluster 1 was the largest one with 28 increased proteins (32 Ksuc sites) that peaked at 6 h during Pi starvation and remained steady thereafter; proteins related to the ribosome, glycolysis/gluconeogenesis, and carbon fixation in photosynthetic organisms pathways were significantly enriched in this cluster. Cluster 2 contained 14 proteins (20 Ksuc sites), which steadily increased during Pi starvation; ribosome- and glycolysis/gluconeogenesis-related proteins were significantly enriched. Cluster 3 contained 25 proteins (28 Ksuc sites), which steadily increased in the first 6 h of Pi starvation, then decreased; ribosome- and oxidative phosphorylation-related proteins were remarkably overrepresented (Figure 6D).

**Figure 7:**
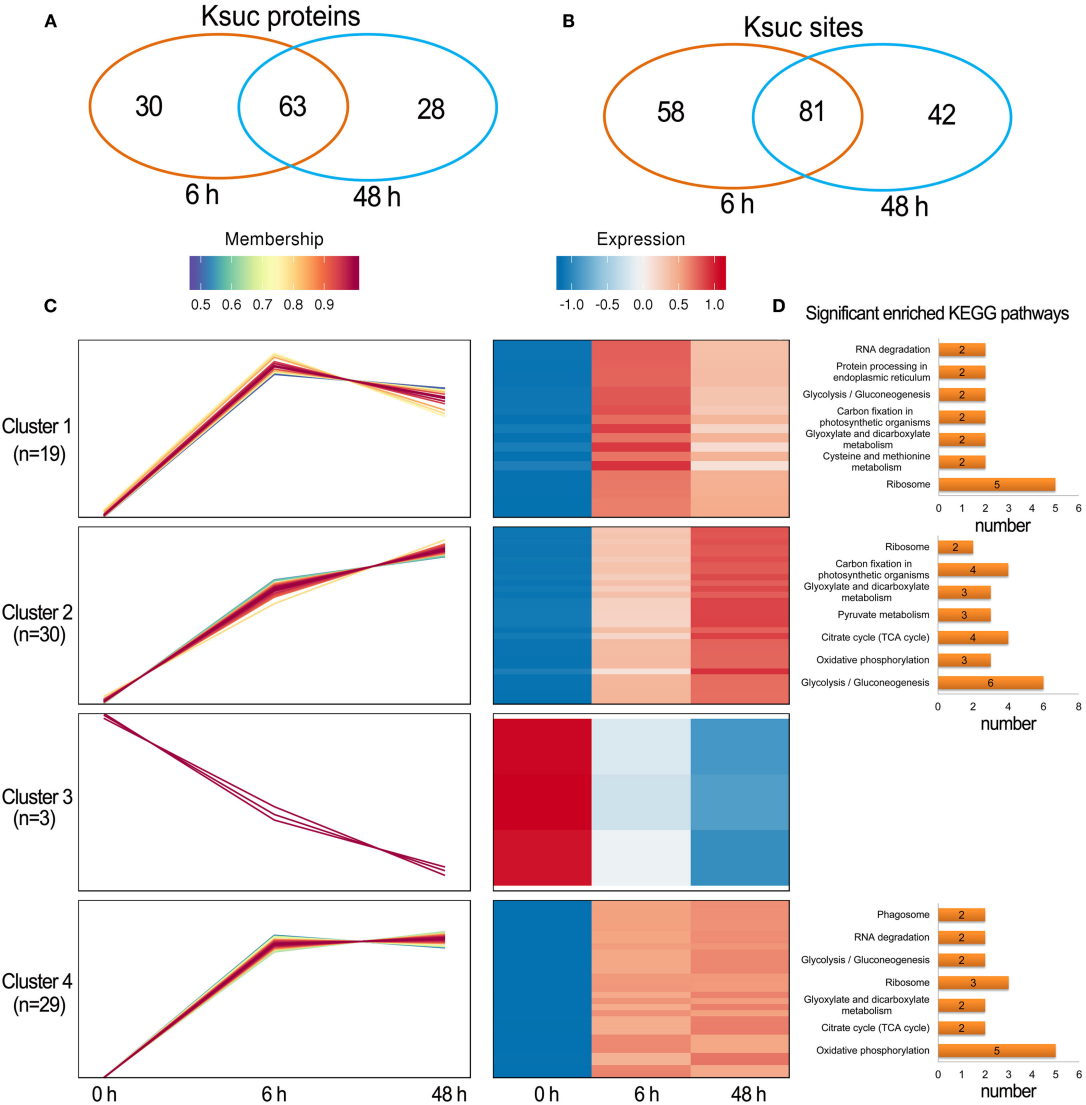
Summary of differentially succinylated proteins in response to Pi recovery. **(A,B)** Venn diagram of difffrentially abundant succinylated proteins (sites) in response to Pi recovery; **(C)** functional clustering analyses (HCL) of the differentially succinylated sites based on the relative succinylation intensity, relative to the control. Cluster identification and number of profiles included in each cluster are indicated on the left. Detailed view of individual profiles is in Supplementary Table 6; **(D)** KEGG pathway enrichment analysis of DFASPs in each cluster.

There were 88 increased DFASPs with 134 succinylation sites and five decreased DFASPs with five succinylation sites, relative to the control, in the Pi recovery (6 h) treatment, and 83 increased DFASPs with 115 succinylation sites and eight decreased DFASPs with eight succinylation sites in the Pi recovery (48 h) treatment (Figure 4D). These DFASPs were mainly enriched in the TCA cycle, glycolysis/gluconeogenesis, and oxidative phosphorylation pathways, which differed from the Pi-starvation phase, except that all were involved in the glycolysis/gluconeogenesis pathway (Supplementary Table 2; Figure 5). The Venn diagram showed 63 increased DFASPs with 81 succinylation sites co-expressed at the stage of Pi deficiency (Pi-recovering DFASPs) (Figures 7A,B) and associated with the glycolysis/gluconeogenesis and TCA cycle pathways (Supplementary Table 5).

The HCL divided Pi-recovering DFASPs into four clusters (Figure 7C; Supplementary Table 6). Cluster 4 was the largest one with 26 increased proteins (29 Ksuc sites) that peaked at 6 h and remained steady thereafter; oxidative phosphorylation and ribosome-related proteins were remarkably overrepresented. Cluster 1 contained 18 proteins (19 Ksuc sites), which steadily increased in the first 6 h, then decreased; ribosome-related proteins were significantly enriched. Cluster 2 contained 24 increased proteins (30 Ksuc sites) that steadily increased during Pi starvation; glycolysis/gluconeogenesis, TCA cycle, and carbon fixation in photosynthetic organisms pathways were notably identified in this cluster. Cluster 3 only contained three proteins and no significant enrichment pathways (Figure 7D).

### Proteome Profiling in Roots Under Pi Starvation and Recovery Conditions

Our analysis yielded 6,734 proteins; of which, 4,920 proteins were precisely quantified with a high degree of repeatability (Supplementary Figure 4). During Pi starvation (6 h), 140 proteins (91 increased and 49 decreased) were differentially abundant (DAPs), and enriched during phenylpropanoid biosynthesis, glycolysis/gluconeogenesis, cysteine and methionine metabolism, and carbon fixation in photosynthetic organisms pathways. During Pi starvation (48 h), only the phenylpropanoid biosynthesis pathway was significantly enriched for the 105 identified DAPs (59 increased and 46 decreased). During Pi recovery (6 h), 78 proteins were differentially abundant (43 increased and 35 decreased) (Supplementary Table 7), which were significantly enriched in the glycolysis/gluconeogenesis, cysteine, and methionine metabolism, MAPK signaling pathways (Supplementary Table 8). During Pi recovery (48 h), the phenylpropanoid biosynthesis, glutathione metabolism, carbon fixation in photosynthetic organisms pathways were significantly enriched in the 88 identified DAPs (44 increased and 44 decreased) (Supplementary Table 7). The KEGG enrichment analysis revealed distinct differences between succinylome and proteome in response to Pi starvation/recovery, although individual processes of both omics were overrepresented (e.g., glycolysis/gluconeogenesis) (Supplementary Table 8). Furthermore, to determine whether the observed changes in succinylation levels were caused by protein abundance changes, we compared the quantified succinylome and proteome. Only four proteins—oxidative phosphorylation (HORVU2Hr1G072660.2) and solute carrier family 25 (HORVU4Hr1G027150.1) in Pi starvation (6 h), and ribulose-bisphosphate carboxylase large chain (HORVU7Hr1G088190.6) and mugineic-acid 3-dioxygenase (HORVU7Hr1G122350.2) in Pi recovery (48 h)—overlapped the DFASPs (Supplementary Table 9), which only accounted for 1.2 and 1.0% of the total DFASPs and DAPs, respectively (Supplementary Figure 5). Therefore, a significant change in succinylated peptides corresponded with proteins that did not significantly change in abundance. This result indicates a very weak correlation between the paired succinylation and protein.

### PPI Networks of Succinylated Proteins in Response to Pi Levels

**Figure 8:**
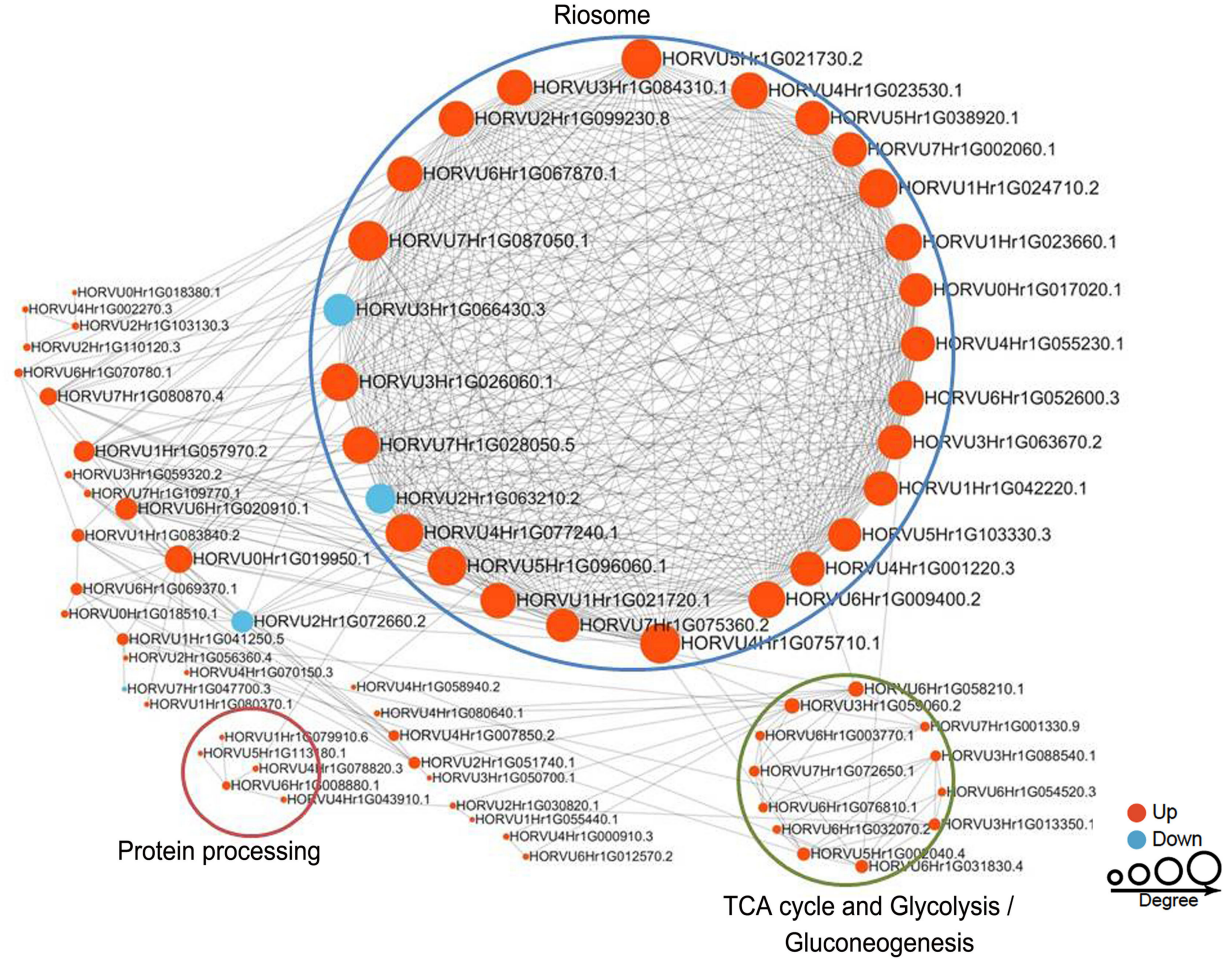
Protein–protein interaction (PPI) network of succinylated proteins in response to Pi starvation. All DFASPs were searched against the STRING database (version 10.5) for PPIs and visualized using Cytoscape (version 3.6.1; http://www.cytoscape.org/). We collated all interactions with a high confidence score (>0.7). A graph-theoretical clustering algorithm, molecular complex detection (MCODE), was used to analyze densely connected regions. The circle size represents the numbers of DFASPs; red indicates increased and blue indicates decreased DFASPs. Further details are in Supplementary Table 10.

**Figure 9:**
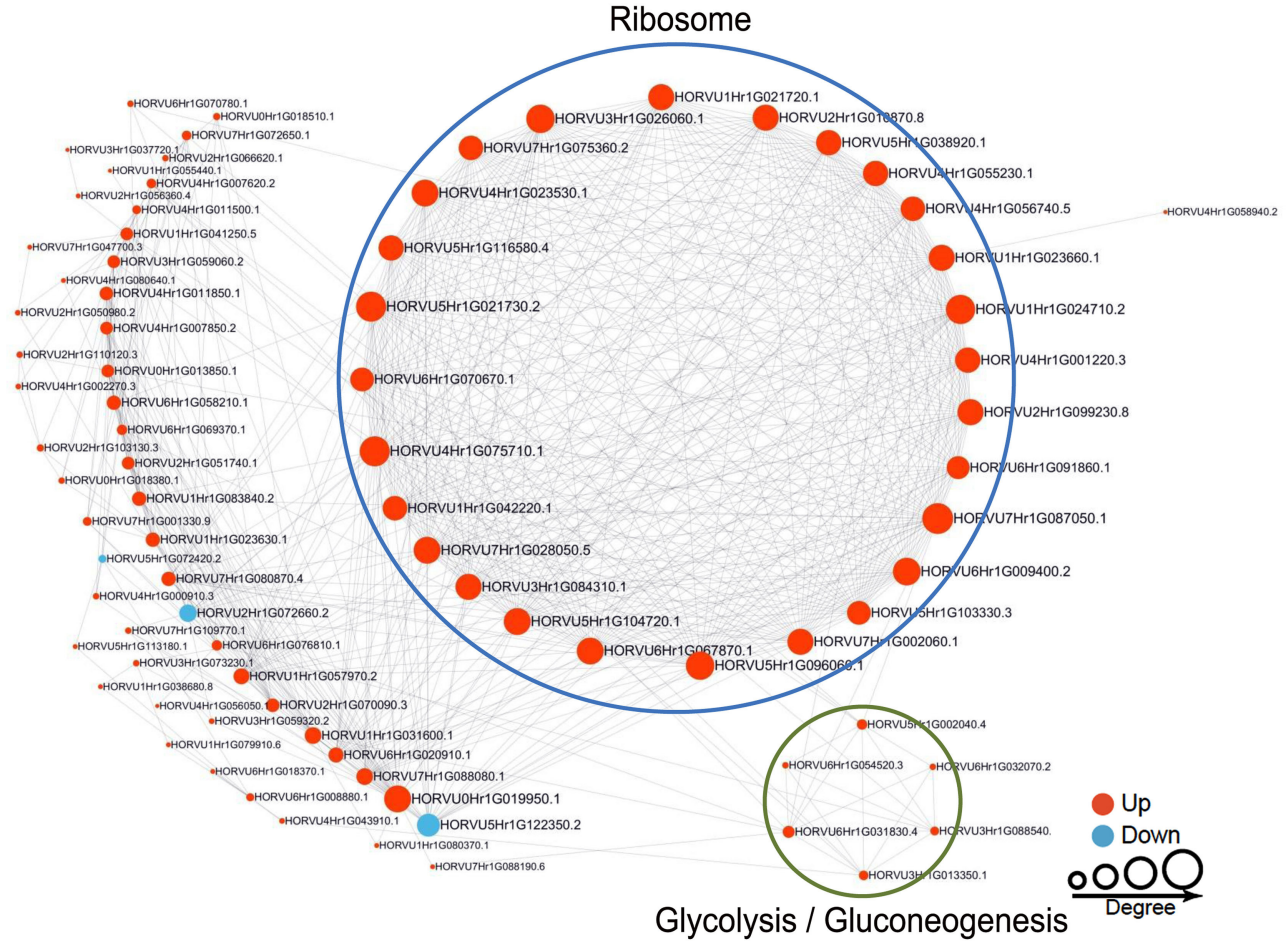
Protein–protein interaction (PPI) network of succinylated proteins in response to Pi recovery. Same as Figure 8.

To reveal the relationships between DFASPs involved in the same biological process, the PPI networks were assembled for all succinylated proteins in roots using Cytoscape software under Pi starvation and recovery. During Pi starvation, the PPI network consisted of 73 DEKSs as nodes, linked by several identified direct physical interactions obtained from the STRING database (Figure 8; Supplementary Table 10). Most of the DFASPs were increased during Pi starvation. The ribosome, TCA cycle, glycolysis/gluconeogenesis, and protein processing pathways were enriched (see highlighted circles of different sizes in Figure 8). In the Pi-recovery phase, 85 DFASPs were nodes, and only three DFASPs decreased, relative to the Pi-starvation phase; proteins with the functional terms “ribosome” and “glycolysis/gluconeogenesis” are highlighted (Figure 9; Supplementary Table 10).

## Discussion

It is well-documented that the high developmental plasticity of plant roots plays an important role in Pi acquisition for coping with adverse environmental conditions (Gruber et al., 2013; Sandhu et al., 2016; Wang et al., 2017; Silva Navas et al., 2019). Plants adjust their RSA to Pi deprivation by inhibiting primary root growth, increasing lateral root density, enhancing root hair development, and forming cluster roots, all of which enhance a plant's soil exploration capacity by increasing the root surface area in the top layers of the soil (Niu et al., 2013; Mora-Macías et al., 2017). In this study, we analyzed the morphology dynamics in barley seedling roots over a time-course of Pi depletion and resupply. Longer exposure to Pi depletion inhibited root development in both barley genotypes—low-Pi-tolerant GN121 and low-Pi-sensitive GN42. Furthermore, GN121 increased root length and lateral root growth more than GN42 during Pi starvation and recovery (Figure 1). These results suggest that the root structure and morphology differ between barley genotypes in response to low-P conditions, and Pi-tolerant GN121 is advantageous for systematic research on root system plasticity as it increases its root surface area in response to P stress.

Several time-course transcriptome (Woo et al., 2012; Secco et al., 2013; Ren et al., 2018), proteome (Iglesias et al., 2013; Jiang et al., 2017), and metabolome (Alexova et al., 2017) studies on Pi depletion and resupply are available. However, PTM studies that focus on temporal development of the Pi-stress response are limited to phosphorylation (Gregory et al., 2009; Yang J. et al., 2019), ubiquitination (Iglesias et al., 2013; Ye et al., 2018; Pan et al., 2019), and sumoylation (Kant et al., 2011; Feng et al., 2017; Datta et al., 2018). Lysine succinylation-a ubiquitous protein PTM pattern-plays a vital role in regulating protein function in both eukaryotic and prokaryotic cells (Xie et al., 2012; Weinert et al., 2013). However, its function in barley, a model plant for *Gramineous* species with tolerance to poor nutrient environments, is largely unknown.

We used antibody-based affinity enrichment, high-resolution LC-MS/MS analysis and integrated bioinformatics analysis to determine whether lysine succinylome (Ksuc) changed in seedling roots of Pi-tolerant GN121 roots under Pi starvation/recovery. An integrated proteomics approach was used to further investigate the changes in succinylome and proteome in barley roots under Pi starvation for 6 h or 48 h, and recovery for 6 or 48 h. To the best of our knowledge, lysine succinylome in barley has not been reported before (Supplementary Table 11). This study identified 2,840 Ksuc sites across 884 proteins with a high score and a high confidence localization score, which is second only to Zhou et al. in their report on rice leaves under oxidative stress (Zhou et al., 2018). This study will greatly expand the knowledge of lysine succinylation substrates and sites in barley roots in response to Pi supply.

### Pathway and Protein-Level Convergence of Succinylation and Abundance Change on Proteins Involved in Pi Stress

Quantitative time-course PTMome analyses identify plant metabolism processes that are regulated under stress conditions (Glen et al., 2019). A systems-level analysis of the root succinylome and proteome response to Pi stress was undertaken to discern protein succinylation levels and/or abundance change in proteins at the pathway and protein level. The KEGG pathway enrichment results of DFASPs and DAPs found that, while some pathways were overrepresented with both omics (e.g., glycolysis/gluconeogenesis), protein succinylation and abundance seem to be divided into distinct metabolic pathways (Figures 6, 7). DFASPs were predominantly involved in the ribosome, glycolysis/gluconeogenesis, TCA cycle, and glyoxylate and dicarboxylate metabolism pathways, while DAPs were involved in phenylpropanoid biosynthesis, glycolysis/gluconeogenesis, and carbon fixation in photosynthetic organisms pathways. At the protein level, only four proteins overlapped between DFASPs and DAPs, which belonged to different metabolic processes (Supplementary Table 9). A quantitative ubiquitylomics analysis of rice seed germination revealed that protein abundance in the ubiquitylome is not correlated with that in the proteome (He et al., 2020). Consistent with this phenomenon, we did not find a significant correlation between the overlapping succinylations and proteins (Supplementary Figure 5). Overall, these two omics mainly intersected on different proteins in the same metabolic pathway in response to Pi stress.

### Motif Comparison of Identified Lysine-Succinylated Peptides

The amino acid residue patterns at particular positions surrounding succinylation lysine revealed a significant bias in eukaryotes and prokaryotes (Park et al., 2013). In *D. officinale*, two types of conserved succinylation motifs, K_+6_Ksuc, and E_−1_Ksuc, were identified (Feng et al., 2017). Four preferred succinylation motifs, P_+1_Ksuc, E_+2_Ksuc, E_−3_KsucK_+1_, and D_+2_ Ksuc, were found in Chinese hickory (*Carya cathayensis*) during the grafting process (Yuan et al., 2019b). Six distinguished succinylation motifs, including K_−6_Ksuc and R_+7_Ksuc, were identified in developing rice seeds (Meng et al., 2019). In our study, consensus peptide motifs for the Ksuc sites were extracted by Motif-X software. Eleven representative Ksuc motifs (K_−10/−9/−8/−7/−6/−5/−4_Ksuc and K_+10/+9/+8/+7_Ksuc) were defined with the preferred amino acid residue of lysine (Figures 4B,C). Similar results were reported in rice leaves exposed to oxidative stress; of the 26 conserved succinylation motifs, one of the K_−10/−9/−8/−7_Ksuc motifs was overrepresented (Zhou et al., 2018). Several representative amino acids, including lysine (K), valine (V), alanine (A), isoleucine (I), glycine (G), and tyrosine (Y), were highly enriched around the succinylated-lysine sites. While the positions differed, these amino acids were not unique but shared with other plants, including *Camellia sinensis* (Xu Y. et al., 2017), rice (Zhou et al., 2018), and *Dendrobium officinale* (Feng et al., 2017). The analysis of conserved motifs and amino acid preferences indicates that protein lysine succinylation is a highly regulated modification process that differs between species, organs, development stages, environmental stimulus, etc.

### Lysine-Succinylated Proteins in Pi Stress

Pi is an essential ingredient in key plant molecules, such as nucleic acids, ATP, and membrane phospholipids (Plaxton and Tran, 2011). Plants improve Pi acquisition and utilization efficiency under Pi deficiency by modulating RSA, regulating the expression and activity of Pi transporters, secreting organic acids and enzymes, and modulating plant metabolic pathways (Pan et al., 2019). To study whether Pi starvation and recovery altered Ksuc, we exposed Pi-tolerant barley seedlings to low Pi for 48 h, followed by Pi recovery for 48 h. Of the identified Ksuc sites with a 1.5-fold change of modified peptides in at least both replicates, relative to the control, about 3.78–4.33% altered their protein abundance in response to Pi stress (Figure 4A), with most (95.24–96.27%) increasing in abundance (Figure 4D). Furthermore, analyzing the subcellular localization of DFASPs revealed that these DFASPs were distributed across diverse cellular components (Supplementary Table 2). However, the KEGG enrichment analysis showed that these DFASPs were only involved in a few metabolic processes, including the ribosome and glycolysis/gluconeogenesis pathways during Pi starvation, and the TCA cycle, glycolysis/gluconeogenesis, and glyoxylate and dicarboxylate metabolism pathways during Pi recovery (Figure 5). These results suggest that barley roots regulate specific Ksuc site changes in response to Pi stress as well as specific metabolic processes. Similar results were obtained in a histone lysine acetylation analysis of rice seedlings during Pi starvation and submergence (Lu et al., 2018). We aimed to extend the current knowledge by focusing on succinylated proteins in the response to and recovery from Pi starvation in plant roots.

### Pi-Responsive DFASPs Regulated During Pi Starvation

The 61 Pi-responsive DFASPs identified in barley roots were significantly increased under Pi-starvation (Supplementary Table 12). We divided these Pi-responsive DFASPs into four main categories, according to the metabolic pathways identified in the KEGG enrichment analysis (*P* < 0.05; Supplementary Table 13). Ribosomal proteins (RPs) are essential components of ribosomes, which are ubiquitous ribonucleoprotein bodies responsible for protein synthesis (Opron and Burton, 2019; Ghulam et al., 2020). Plant cells regulate protein synthesis in response to nutrients and stress by controlling RP expression (Szakonyi and Byrne, 2011; Karunadasa et al., 2020). In this study, the most prominent cluster was related to ribosome function (Figure 8) and enriched in the PPI network, suggesting an important role of ribosome biogenesis/translation in the Pi-starvation response. Expression of the 40S ribosomal protein S6 (PRS6) gene was induced by low temperature in soybean (Kim et al., 2004). The 30S ribosomal protein S9 (RPS9) plays a crucial role in ribosome biogenesis and normal cell growth and proliferation (Qiu et al., 2018). The 60 S ribosomal protein L6 (RPL6) directly interacts with histone H2A and regulates the DNA damage response (Yang C. et al., 2019), and ribosomal protein S4 (RPS4) playing a regulatory role in ribosomal RNA operon antitermination (Torres et al., 2001). Protein processing-related proteins, especially those for regulating transcription, such as elongation factor 1-alpha (EEF1A1), elongation factor Ts, and eukaryotic translation initiation factor 5A-1 (EIF5A), also increased in the succinyltome. Several glycolysis and TCA cycle metabolism-related enzymes, including phosphoglycerate kinase (PGK), enolase, isocitrate dehydrogenase (IDH), dihydrolipoyllysine-residue acetyltransferase component of pyruvate dehydrogenase complex, mitochondrial (DLAT), and pyruvate dehydrogenase E1 component subunit alpha (PDHA1) were identified as DFASPs (Supplementary Table 12). Notably, the pyruvate dehydrogenase complex catalyzes the overall conversion of pyruvate to acetyl-CoA and CO_2_, thereby linking the glycolytic pathway to the TCA cycle. Succinylation is also involved in oxidative phosphorylation metabolism coupled to ATP synthesis through an electrochemical transmembrane gradient (Yang and Gibson, 2019). Increases in the expression of ATPase subunits have been related to the need for more ATP synthase to export protons out of cells (Rott et al., 2011). Our results showed that β (AtpB), d (AtpB) subunits of FoF1-ATPase and NDH-1 subunit F, and NADH-ubiquinone oxidoreductase 75 kD subunit, mitochondrial, V-type proton ATPase subunit E, V-type ATP synthase alpha chain increased Ksuc levels under Pi starvation. Proteins related to other metabolic pathways, such as carbon fixation in photosynthetic organisms, glyoxylate, and dicarboxylate metabolism pathways, were identified in DFASPs. Of these, 17 DFASPs did not respond to Pi recovery (Supplementary Table 14), and were mainly involved in protein processing, glycolysis, and TCA cycle pathways. Most notably, the succinylation level of protein processing, especially 60S ribosomal protein L4-1(RPL4), was increased 7.21- and 6.91-fold during Pi starvation at 6 h and 48 h, respectively (Supplementary Table 14).

### Pi-Responsive DFASPs Regulated During Pi Recovery

**Figure 10:**
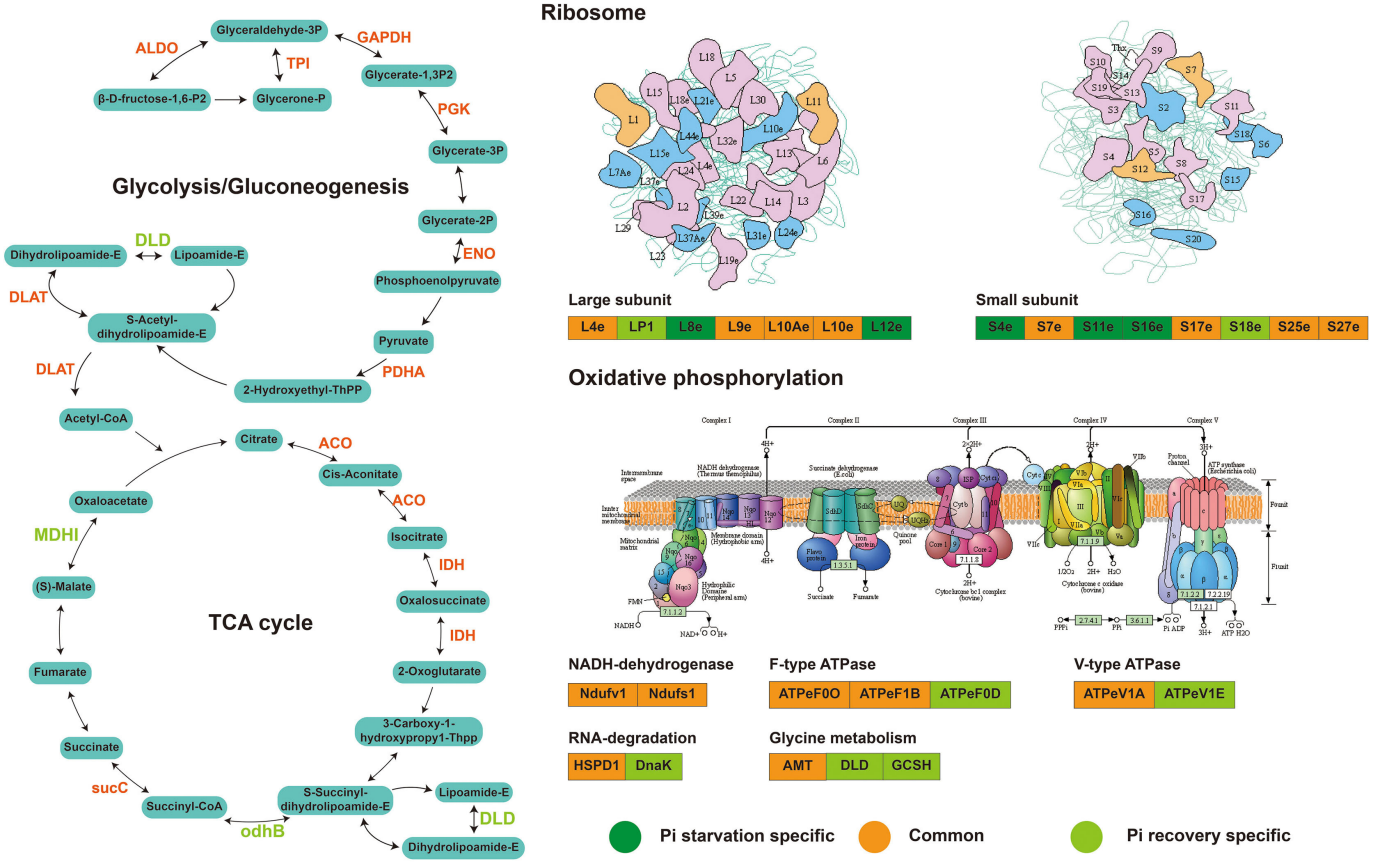
Major succinylation-mediated metabolic processes and proteins involved in short-term Pi starvation and recovery, as depicted by succinyl-proteome analyses. For details of the proteins and their abbreviations, see Supplementary Table 14.

We identified 63 succinylated proteins that were co-expressed differentially in roots during Pi recovery (Supplementary Table 12) and divided into four main categories with the KEGG enrichment metabolic pathway (*P* < 0.05; Supplementary Table 13). Forty-four DFASPs overlapped during Pi starvation. Similar to Pi starvation, the metabolic pathways of the TCA cycle, glycolysis/gluconeogenesis, glyoxylate and dicarboxylate metabolism, and oxidative phosphorylation were prominently represented (Supplementary Table 13). Among these DFASPs, 18 did not respond to Pi starvation (Supplementary Table 14) and are mainly involved in protein processing, glycolysis, and TCA cycle pathways. Especially, the succinylation level of a protein disulfide-isomerase (PDR) of protein processing, whose major function is to catalyze disulfide bond formation in newly synthesized proteins and respond to biotic (Li et al., 2020) and abiotic (Hashimoto and Komatsu, 2007) stresses, was increased 3.78-fold during Pi recovery (6 h) (Supplementary Table 14). Overall, analysis of the succinylated proteins in response to Pi starvation and recovery revealed that the DFASPs involved in metabolic pathways during Pi starvation differed from those during Pi recovery. Hence, this study focused on characterizing the succinylated protein response to and recovery from Pi starvation (Figure 10). The biological function and functional interplay among succinylated proteins in response to Pi starvation requires further investigation.

## Conclusion

Specific PTMs control the structure and function of proteins that respond to the environment and metabolic stimuli. In this study, we presented the plant regulation processes of protein succinylation in roots in response to and recovery from Pi starvation by profiling the dynamic succinylome and proteome in a Pi-tolerant barley genotype (GN121). We identified 2,840 Ksuc sites across 884 proteins. The Ksuc motifs are preferred amino acid residue of lysine, and protein lysine succinylation is a highly regulated modification process. Of these, 61 and 62 increased succinylated proteins were co-expressed during Pi starvation and Pi recovery, respectively. These Pi- responsive succinylated proteins that regulate Pi starvation and recovery mainly involved Pi starvation metabolic pathways. Taken together, this study provides essential data resources for exploring the roles of Ksuc in regulating plant root responses to Pi starvation, including protein processing, glycolysis, and TCA pathways, and has extended our knowledge on important, yet relatively poorly characterized, PTMs of succinylation in plants.

## Data Availability Statement

The original contributions presented in the study are publicly available. This data can be at: The mass spectrometry data of the succinylome and proteome have been deposited at the ProteomeXchange with dataset identifier PXD022052 and PXD022053, respectively.

## Author Contributions

JW, ZM, CL, and PR carried out the proteomic analysis and drafted the manuscript. LY, BL, YM, and XM participated in material culture and performed the statistical analysis. HW and XS conceived of the study and participated in its design. HW, ES, and KY helped to draft the manuscript. All authors read and approved the final manuscript.

## Conflict of Interest

The authors declare that the research was conducted in the absence of any commercial or financial relationships that could be construed as a potential conflict of interest.

## Supplementary Material

Supplementary Table 1 Statistics of the differentially succinylated sites and proteins under Pi starvation (6, 48 h) and Pi recovery (6, 48 h).

Supplementary Table 2 KEGG pathway enrichment analysis of DFASPs in roots under Pi starvation (6, 48 h) and Pi recovery (6, 48 h).

Supplementary Table 3 GO enrichment analysis of DFASPs in roots under Pi starvation (6, 48 h) and Pi recovery (6, 48 h).

Supplementary Table 4 InterPro domain enrichment of DFASPs in roots under Pi starvation (6, 48 h) and Pi recovery (6, 48 h).

Supplementary Table 5 KEGG pathway enrichment analysis of co-expressed DFASPs in roots under Pi starvation (6, 48 h) and Pi recovery (6, 48 h).

Supplementary Table 6 Cluster identification and number of differentially succinylated proteins in response to Pi starvation and recovery.

Supplementary Table 7 Statistics of DFASPs under Pi starvation (6, 48 h) and Pi recovery (6, 48 h).

Supplementary Table 8 KEGG pathway enrichment analysis of DAPs in roots under Pi starvation (6, 48 h) and Pi recovery (6, 48 h).

Supplementary Table 9 Overlap of identified DAPs with DFASPs in roots in response to Pi stress.

Supplementary Table 10 Overview of the succinylated proteins used to construct the protein–protein interaction (PPI) network in response to Pi starvation and Pi recovery.

Supplementary Table 11 Lysine succinylation analysis in different plant species.

Supplementary Table 12 Statistics of DFASPs that were co-expressed differentially in roots during Pi starvation and recovery.

Supplementary Table 13 KEGG pathway enrichment analysis of DFASPs in roots under Pi starvation and recovery.

Supplementary Table 14 List of major succinylation-mediated proteins involved in short-term Pi starvation and recovery.

Supplementary Figure 1 Biological process enrichment analysis of DFASPs in roots under Pi starvation (6, 48 h) and Pi recovery (6, 48 h).

Supplementary Figure 2 Subcellular localization of DFASPs in roots under Pi starvation (6, 48 h) and Pi recovery (6 and 48 h).

Supplementary Figure 3 InterPro domain enrichment of DFASPs in roots under Pi starvation (6, 48 h) and Pi recovery (6, 48 h) (*P* < 0.05). Circle size represents the number of succinylated proteins within the domain family. Colors represent the significance of enrichment.

Supplementary Figure 4 Summary of DAPs in response to Pi starvation and recovery. **(A)** Summary of the proteins identified and quantified; **(B)** overview of DAPs (fold change > 1.5, *P* < 0.05); **(C)** KEGG pathway enrichment analysis of DAPs in roots during Pi starvation (6, 48 h); **(D)** KEGG pathway enrichment analysis of DAPs in roots during Pi recovery (6, 48 h).

Supplementary Figure 5 Venn diagram showing the number of proteins overlapping between DFASPs and DAPs in roots in response to Pi stress.
