# Supplementary material for: Dynamic Responses of Barley Root Succinyl-Proteome to Short-Term Phosphate Starvation and Recovery
Source: Front Plant Sci. 2021 Mar 31;12:649147. doi: 10.3389/fpls.2021.649147 (PMC8045032; doi:10.3389/fpls.2021.649147)
Supplement: Supplementary Table 1 — Statistics of the differentially succinylated sites and proteins under Pi starvation (6, 48 h) and Pi recovery (6, 48 h). [file Data_Sheet_1.ZIP › Additional files/Tables/Table S11.docx]

**Table S11 Lysine succinylation analysis in different plant species**

| Species | Protein | Sites | Organ | Year | reference |
| --- | --- | --- | --- | --- | --- |
| *Patchouli* | 493 | 1097 | leaf | 2019 | Global analysis of lysine succinylation in *patchouli* plant leaves |
| Rice | 347 | 854 | seed | 2019 | Comprehensive analysis of the lysine succinylome and protein co-modifications in developing rice seeds |
| *Paulownia tomentosa* | 1270 | 1970 | seeding | 2019 | Phytoplasma-induced changes in the acetylome and Succinylome of *paulownia tomentosa* provide evidence for Involvement of acetylated proteins in Witches' Broom disease |
| Rice | 1,024 | 2,593 | Leaf | 2018 | Oxidative stress-triggered interactions between the succinyl-and acetyl-proteomes of rice leaves |
| Tomato (*Solanum lycopersicum*) | 202 | 347 | micro-Tom | 2016 | Proteome-wide identification of lysine succinylation in the proteins of tomato (solanum lycopersicum) |
| Chinese hickory (*Carya cathayensis*) | 259 | 202 | plants | 2019 | Quantitative succinyl-proteome profiling of Chinese hickory (*Carya cathayensis*) during the grafting process |
| Tea | 86 | 142 | leaf | 2020 | A qualitative proteome-wide lysine succinylation profiling of tea revealed its involvement in primary metabolism |
| *Brachypodium distachyon* L. | 353 | 605 | leaf | 2016 | First comprehensive proteome analyses of lysine acetylation and succinylation in seedling leaves of *Brachypodium distachyon* L. |
| Common wheat | 173 | 330 | leaf | 2017 | Global analysis of protein lysine succinylation profiles in common wheat |
| Tea [*Camellia sinensis* (L.) *O. Kuntze*] | 2132 | 3530 | leaf | 2017 | Quantitative succinyl-proteome profiling of *Camellia sinensis* cv.'Anji Baicha'during periodic albinism |
| Rice | 346 | 710 |  | 2019 | Global proteomic analysis reveals widespread lysine succinylation in rice seedlings |
| *Ananas comosus var. bracteatus* | 335 | l, 855 | Leaf | 2020 | Systematic identification and comparative  analysis of lysine succinylation between the  green and white parts of chimeric leaves of  *Ananas comosus var. bracteatus* |
| rice | 261 | 665 | seed | 2016 | Global proteome analyses of lysine acetylation and succinylation reveal the widespread involvement of both modification in metabolism in the embryo of germinating rice seed |
| Taxus | 193 | 325 | bark | 2016 | Succinyl-proteome profiling of a high taxol containing hybrid Taxus species (Taxus × media) revealed involvement of succinylation in multiple metabolic pathways |
