# Supplementary figures and images for: Dynamic Responses of Barley Root Succinyl-Proteome to Short-Term Phosphate Starvation and Recovery
Source: Front Plant Sci. 2021 Mar 31;12:649147. doi: 10.3389/fpls.2021.649147 (PMC8045032; doi:10.3389/fpls.2021.649147)

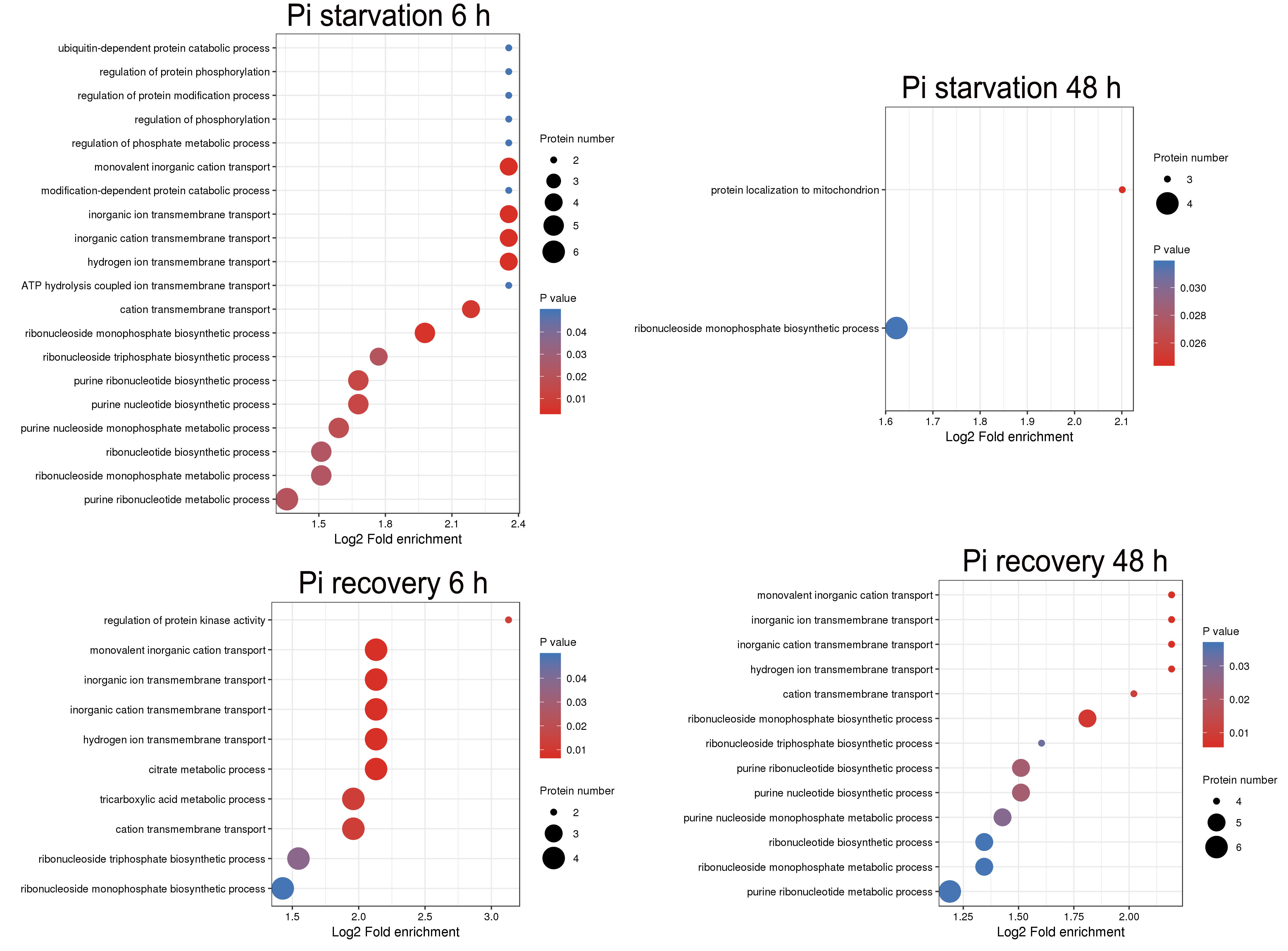

Supplement: Supplementary Table 1 — Statistics of the differentially succinylated sites and proteins under Pi starvation (6, 48 h) and Pi recovery (6, 48 h). [file Data_Sheet_1.ZIP › Additional files/Figures/Figure S1.jpg]

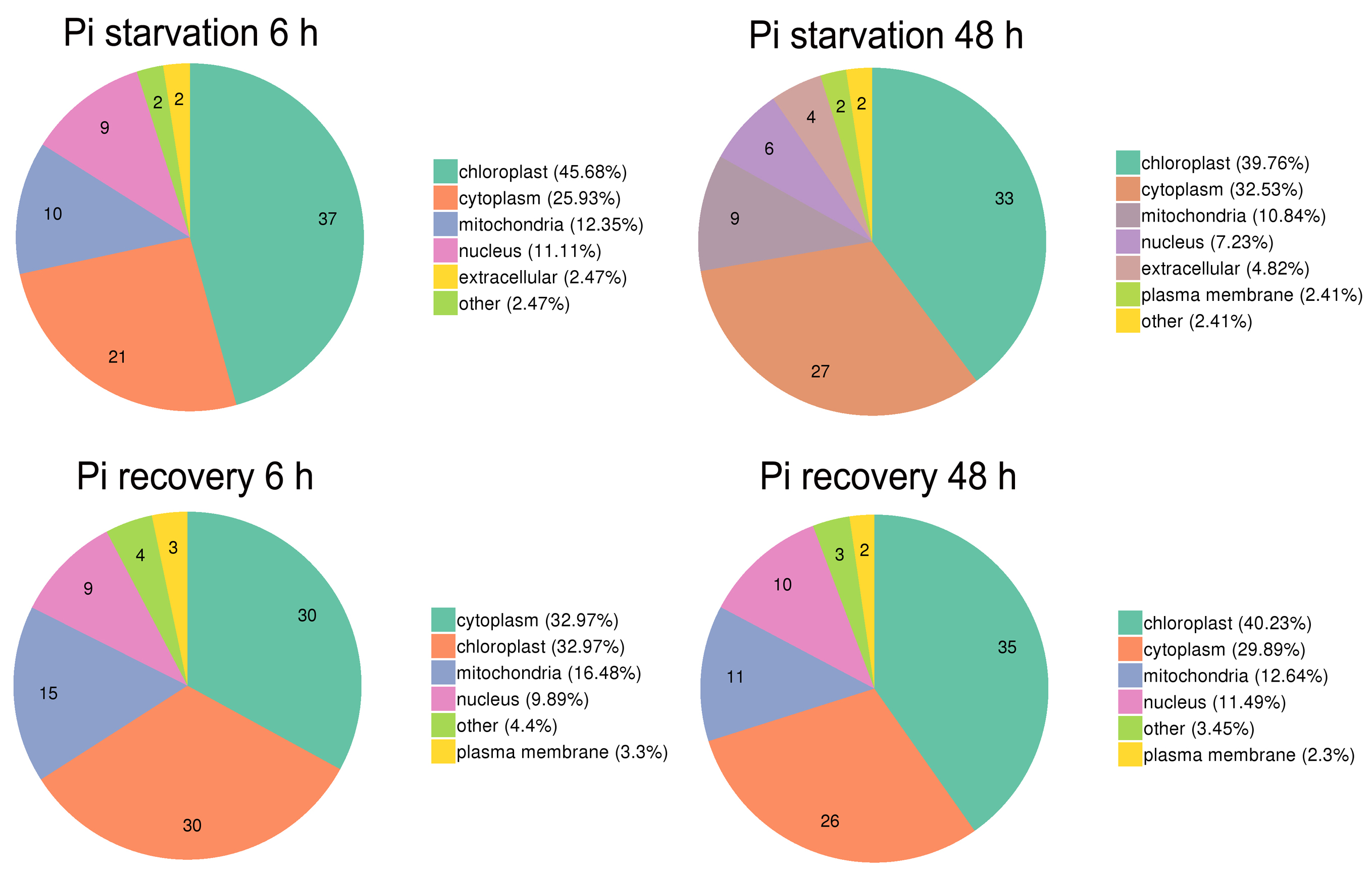

Supplement: Supplementary Table 1 — Statistics of the differentially succinylated sites and proteins under Pi starvation (6, 48 h) and Pi recovery (6, 48 h). [file Data_Sheet_1.ZIP › Additional files/Figures/Figure S2.jpg]

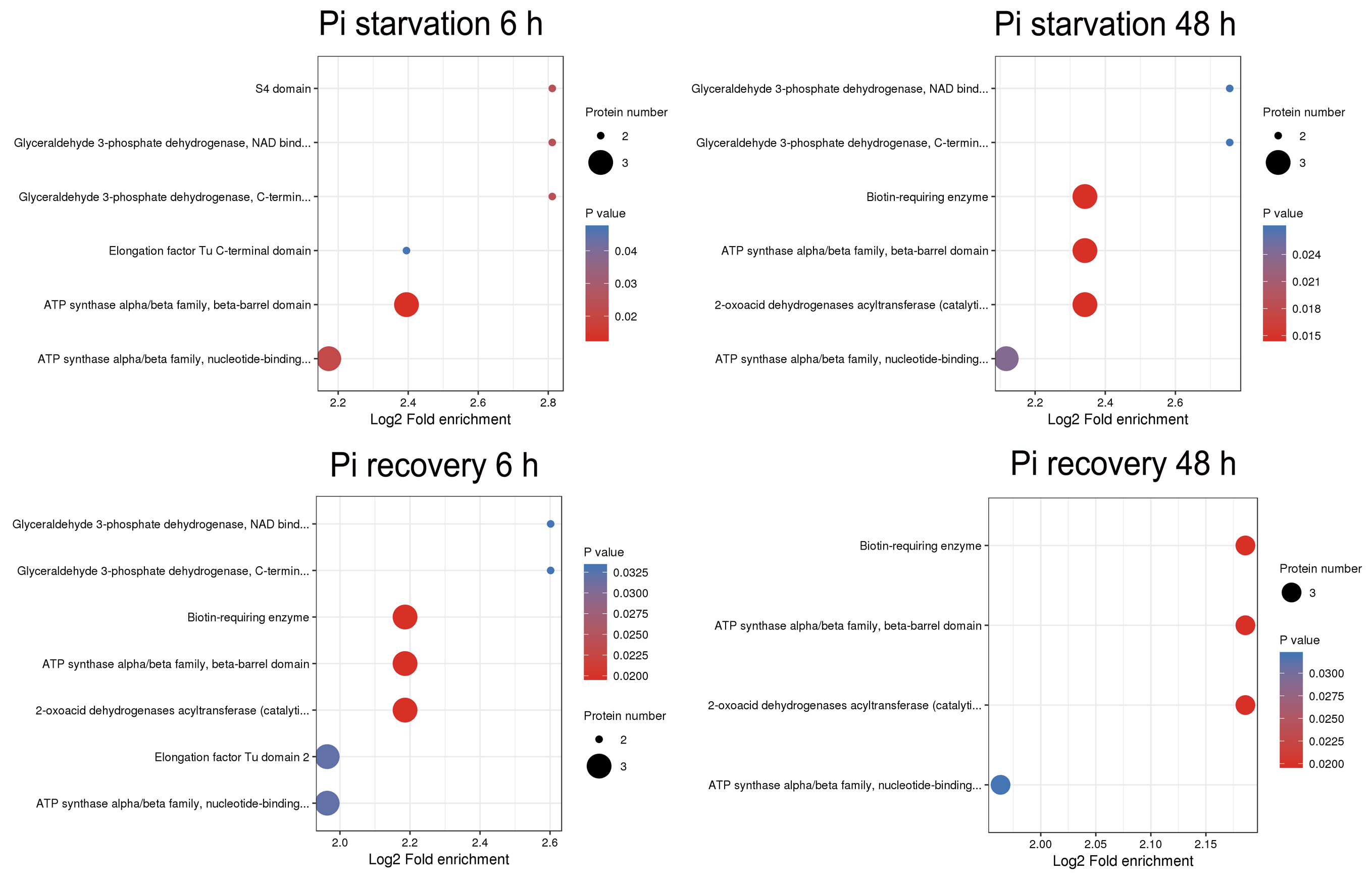

Supplement: Supplementary Table 1 — Statistics of the differentially succinylated sites and proteins under Pi starvation (6, 48 h) and Pi recovery (6, 48 h). [file Data_Sheet_1.ZIP › Additional files/Figures/Figure S3.jpg]

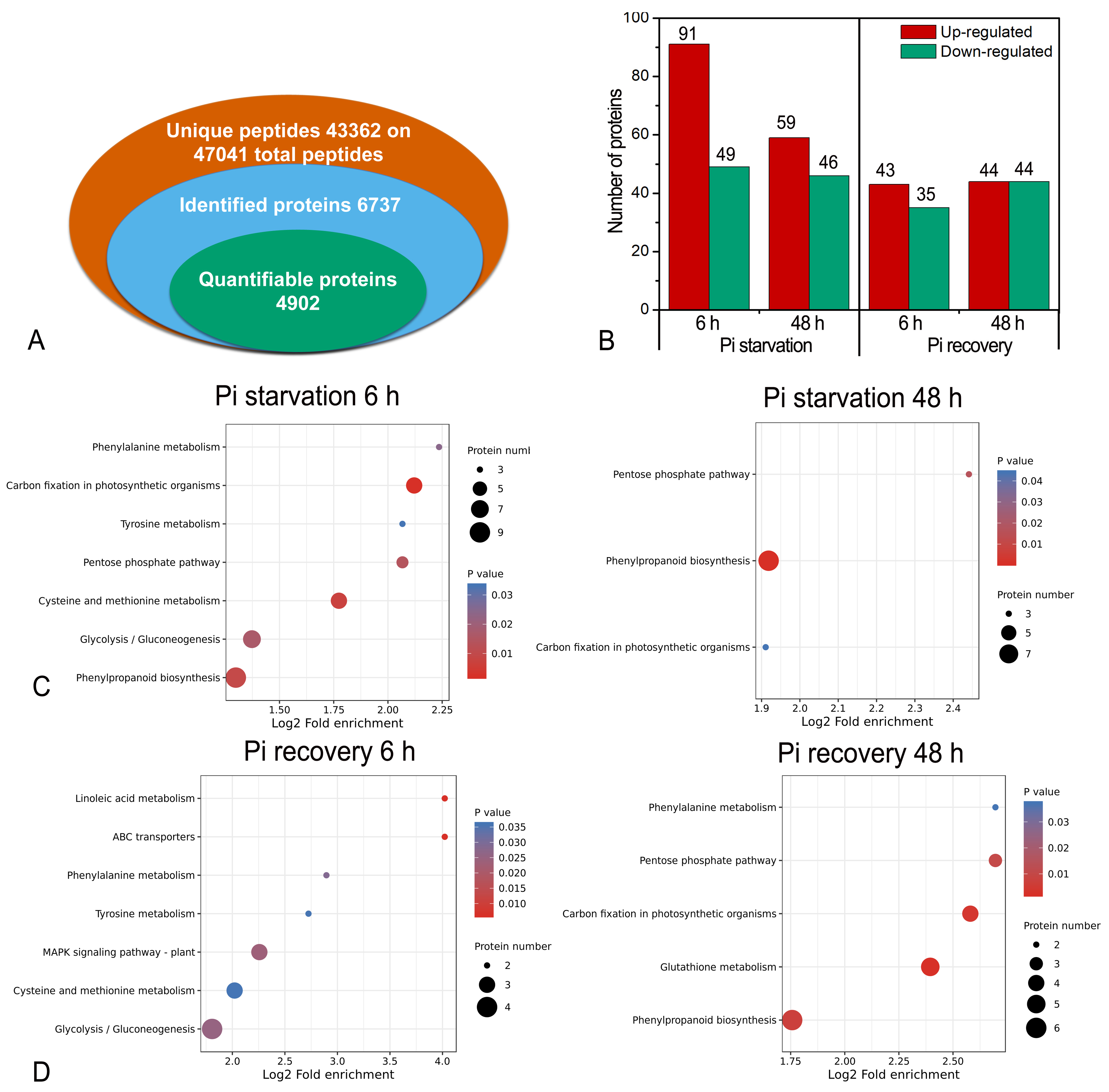

Supplement: Supplementary Table 1 — Statistics of the differentially succinylated sites and proteins under Pi starvation (6, 48 h) and Pi recovery (6, 48 h). [file Data_Sheet_1.ZIP › Additional files/Figures/Figure S4.jpg]

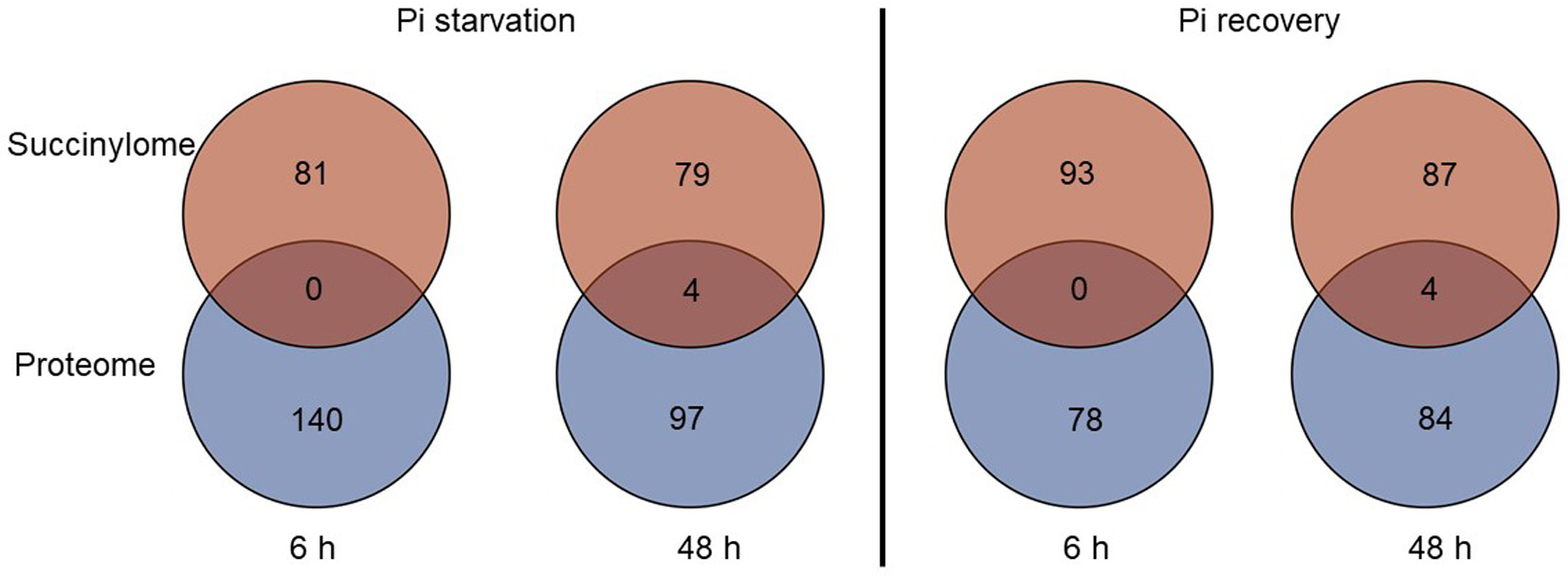

Supplement: Supplementary Table 1 — Statistics of the differentially succinylated sites and proteins under Pi starvation (6, 48 h) and Pi recovery (6, 48 h). [file Data_Sheet_1.ZIP › Additional files/Figures/Figure S5.jpg]
